# Supplementary material for: Identification of endothelial and mesenchymal FOXF1 enhancers involved in alveolar capillary dysplasia
Source: Nat Commun. 2024 Jun 19;15:5233. doi: 10.1038/s41467-024-49477-6 (PMC11187179; doi:10.1038/s41467-024-49477-6)
Supplement: Supplementary file 1 — Supplementary Information [file 41467_2024_49477_MOESM1_ESM.pdf]

Supplementary Materials

Identification of Endothelial and Mesenchymal FOXF1 Enhancers Involved in Alveolar Capillary Dysplasia

Guolun Wang[1,2*], Bingqiang Wen[3], Minzhe Guo[1,2], Enhong Li[3], Yufang Zhang[1], Jeffrey A. Whitsett[1,2], Tanya V. Kalin[3], Vladimir V. Kalinichenko[3,4*]

[1]*Division of Pulmonary Biology and Neonatology, Perinatal Institute, Cincinnati Children's Research Foundation, Cincinnati, OH*

[2]*Department of Pediatrics, University of Cincinnati College of Medicine, Cincinnati, OH*

[3]*Phoenix Children's Research Institute, Department of Child Health, University of Arizona, College of Medicine, Phoenix, AZ*

[4]*Division of Neonatology, Phoenix Children's Hospital, Phoenix, AZ*

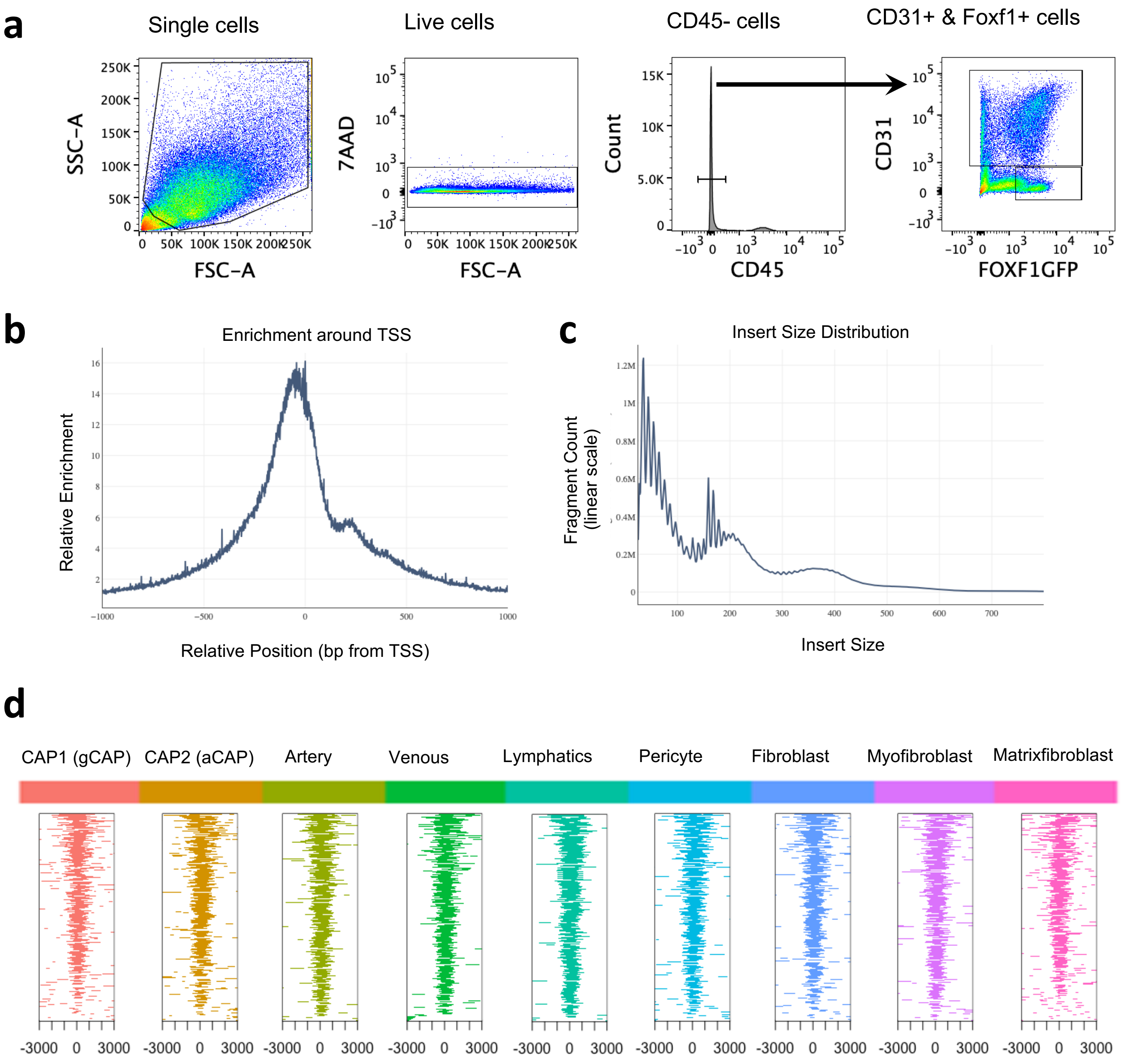

a
Single cells
Live cells
CD45- cells
CD31+ & Foxf1+ cells
SSC-A
FSC-A
7AAD
Count
CD45
CD31
FOXF1GFP
b
Enrichment around TSS
Relative Enrichment
Relative Position (bp from TSS)
c
Insert Size Distribution
Fragment Count (linear scale)
Insert Size
d
CAP1 (gCAP)
CAP2 (aCAP)
Artery
Venous
Lymphatics
Pericyte
Fibroblast
Myofibroblast
Matrixfibroblast

Supplementary Figure 1. The experimental design and quality control of multiome library.

a, The flow cytometry strategy shows the isolation of Foxf1-positive single cells from mouse E18.5 lungs. **b,** The transcriptional start site (TSS) profile shows a high degree of chromatin accessibility compared to the flanking regions of transcription start site. **c,** The insert size distribution transposase-accessible fragments displays a typical sawtooth pattern which indicates the periodicity of ~150 bp that correspond to the number of nucleosomes, and the transposase-accessible fragments span (nucleosome free, mononucleosome, and dinucleosome fragments). **d,** The heatmaps show accessible peaks binding to TSS regions in different cell types.

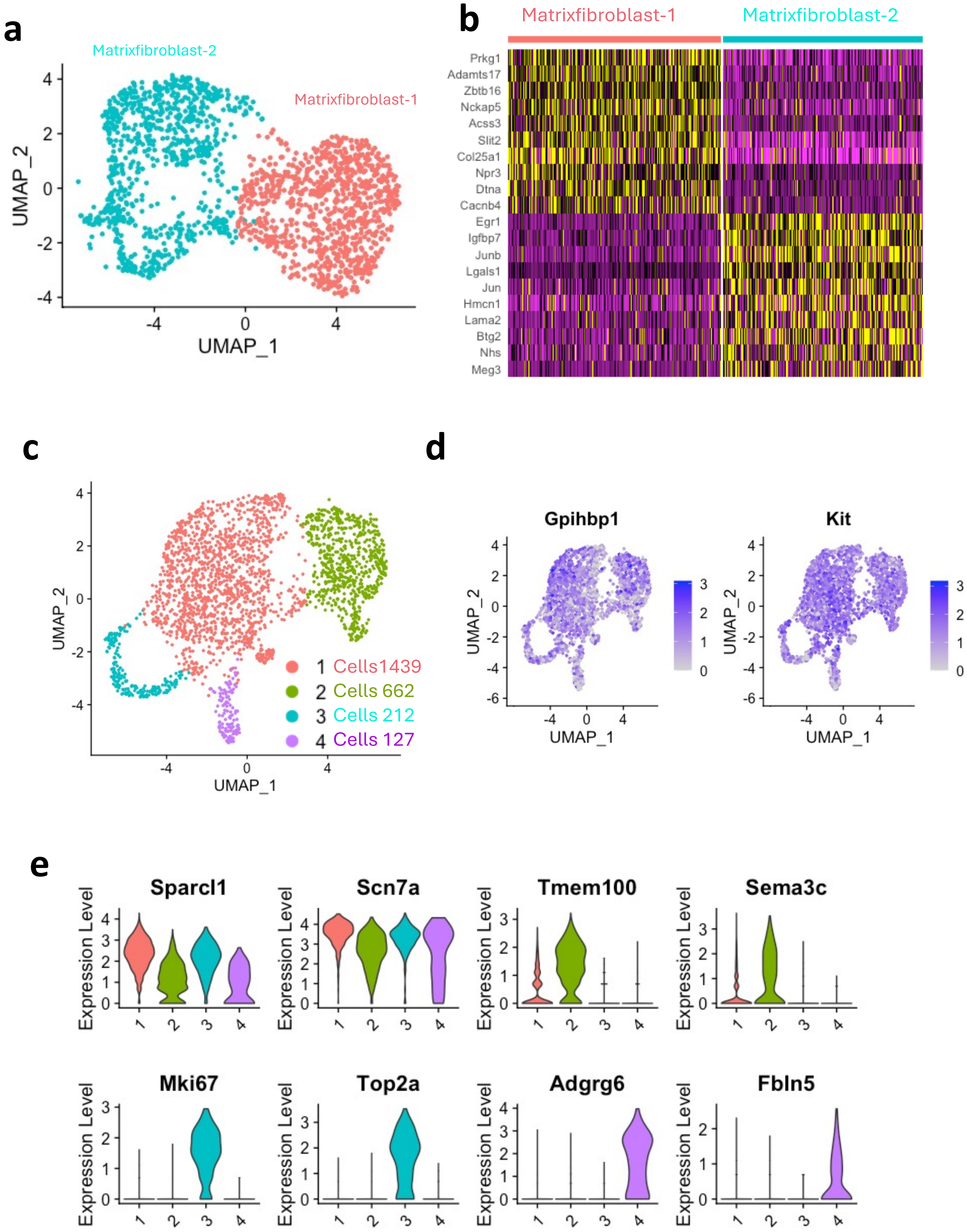

a
Matrixfibroblast-2
Matrixfibroblast-1
UMAP_2
UMAP_1
b
Matrixfibroblast-1
Matrixfibroblast-2
Prkg1
Adamts17
Zbtb16
Nckap5
Acss3
Slit2
Col25a1
Npr3
Dtna
Cacnb4
Egr1
Igfbp7
Junb
Lgals1
Jun
Hmcn1
Lama2
Btg2
Nhs
Meg3
c
1 Cells1439
2 Cells 662
3 Cells 212
4 Cells 127
UMAP_2
UMAP_1
d
Gpihbp1
Kit
UMAP_2
UMAP_1
e
Sparcl1
Scn7a
Tmem100
Sema3c
Mki67
Top2a
Adgrg6
Fbln5
Expression Level

Supplementary Figure 2. Sub-clustering of matrix fibroblasts and gCAP cells.

a, Sub-clustering of matrix fibroblasts indicates that these cells consist of two cell clusters. **b,** The heatmap shows representative markers enriched in each cell sub-cluster. **c,** Sub-clustering of CAP1 (gCAP) cells indicates that these cells consist of four distinct cell clusters: sub-clusters 1, 2, 3 and 4. Sub-cluster 1 contains the majority of CAP1 cells (~60%). **d,** All CAP1 cells express *Gpihbp1* and *Kit* that are well-defined markers for CAP1. **e,** Violin plots show that sub-cluster 1 expresses high levels of *Scn7a* and *Sparcl1*. Sub-cluster 2 expresses high levels of *Tmem100* and *Sema3c*. Sub-cluster 3 is a highly proliferative cell subset as shown by the enrichment of *Mki67* and *Top2a*. *Adgrg6* and *Fbln5* mRNAs are selectively expressed in sub-cluster 4.

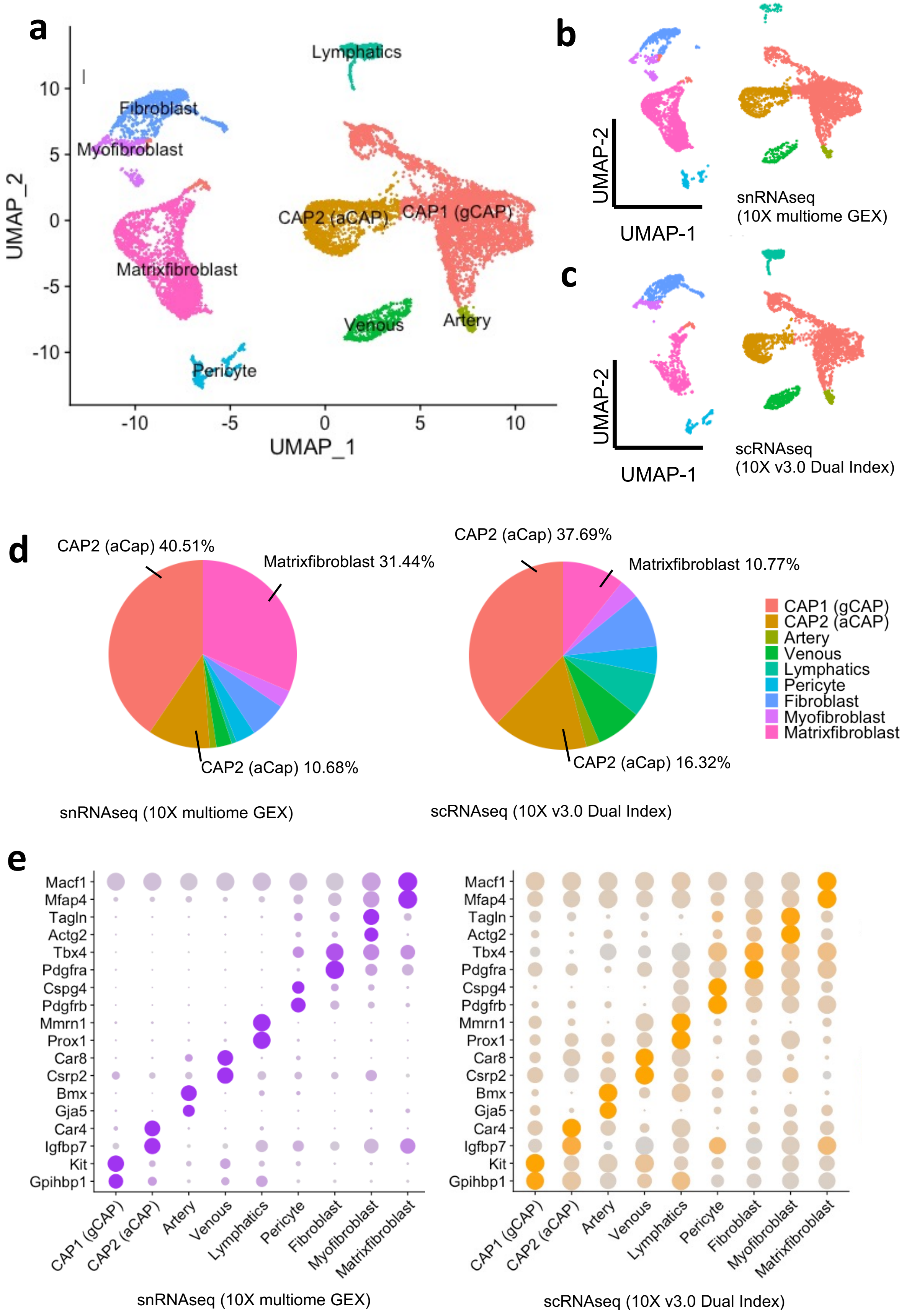

a
Lymphatics
Fibroblast
Myofibroblast
CAP2 (aCAP)
CAP1 (gCAP)
Matrixfibroblast
Venous
Artery
Pericyte
UMAP_2
UMAP_1
b
UMAP-2
UMAP-1
snRNAseq
(10X multiome GEX)
c
UMAP-2
UMAP-1
scRNAseq
(10X v3.0 Dual Index)
d
CAP2 (aCap) 40.51%
Matrixfibroblast 31.44%
CAP2 (aCap) 10.68%
snRNAseq (10X multiome GEX)
CAP2 (aCap) 37.69%
Matrixfibroblast 10.77%
CAP2 (aCap) 16.32%
scRNAseq (10X v3.0 Dual Index)
CAP1 (gCAP)
CAP2 (aCAP)
Artery
Venous
Lymphatics
Pericyte
Fibroblast
Myofibroblast
Matrixfibroblast
e
Macf1
Mfap4
Tagln
Actg2
Tbx4
Pdgfra
Cspg4
Pdgfrb
Mmrn1
Prox1
Car8
Csrp2
Bmx
Gja5
Car4
Igfbp7
Kit
Gpihbp1
CAP1 (gCAP)
CAP2 (aCAP)
Artery
Venous
Lymphatics
Pericyte
Fibroblast
Myofibroblast
Matrixfibroblast
snRNAseq (10X multiome GEX)
scRNAseq (10X v3.0 Dual Index)

Supplementary Figure 3. Integration of single-cell RNAseq and single-nuclei GEX library from multiome sequencing.

a, The integrated projection of single-cell RNAseq library (10X v3 3’ dual index) and single-nuclei GEX (RNA or gene expression) library from multiome sequencing. Both libraries were prepared from lungs of E18.5 embryos that were enzymatically digested to obtain single cell suspensions. GFP-expressing cells were FACS-sorted from *Foxf1-GFP* reporter mouse line. Cell clusters were identified using unsupervised clustering UMAP projection. **b,** The UMAP embedding of single-nuclei RNA sequencing (10X multiome GEX library). **c,** The UMAP embedding of single-cell RNA sequencing (10X v3 chemistry dual index library). **d,** Pie-plots compare the cellular distribution from 10X multiome GEX library and 10X v3 dual index library. **e,** Dot-plots show the consistency of clustering between the two libraries when cell cluster-specific markers are compared.

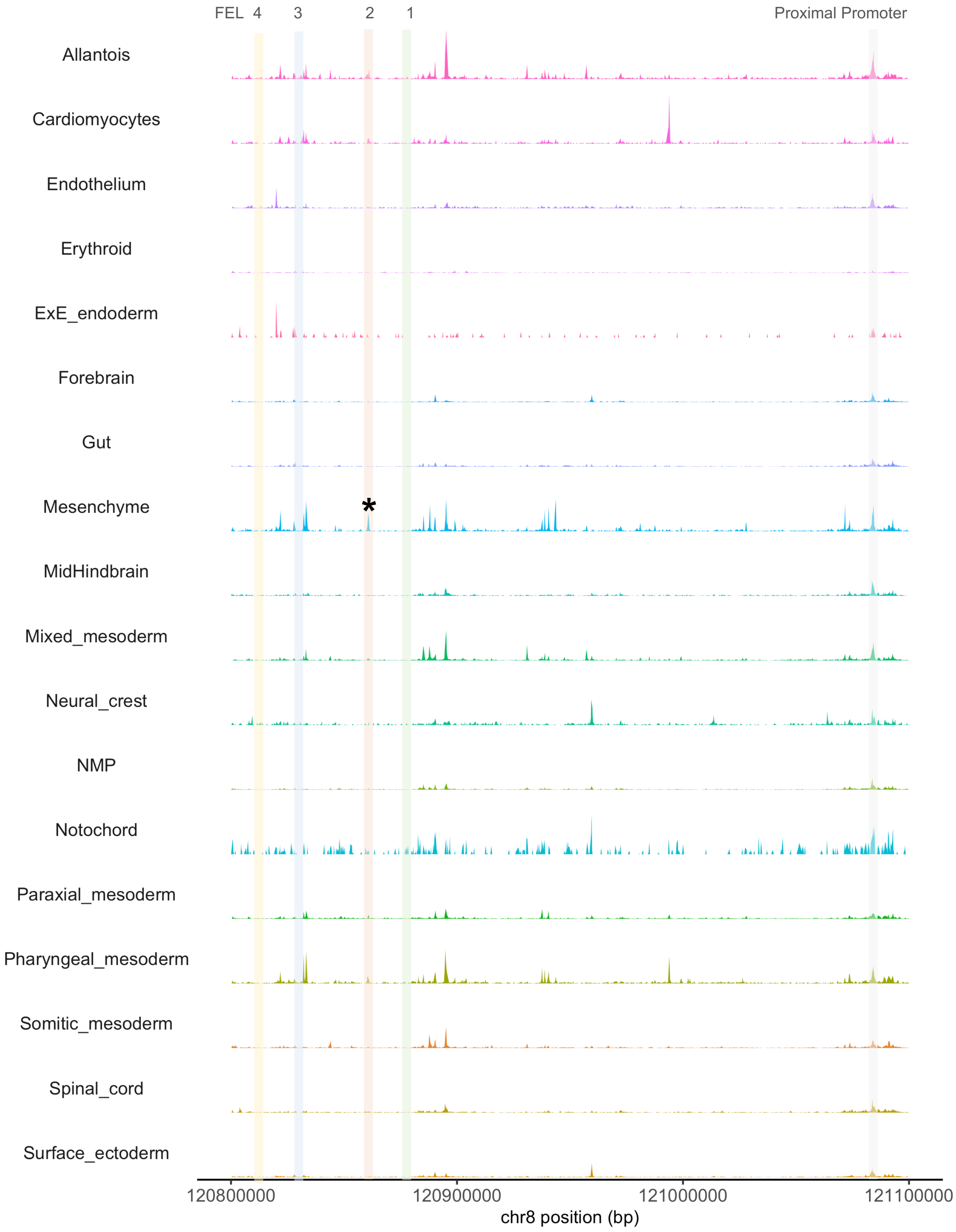

FEL
4
3
2
1
Proximal Promoter
Allantois
Cardiomyocytes
Endothelium
Erythroid
ExE_endoderm
Forebrain
Gut
Mesenchyme
*
MidHindbrain
Mixed_mesoderm
Neural_crest
NMP
Notochord
Paraxial_mesoderm
Pharyngeal_mesoderm
Somitic_mesoderm
Spinal_cord
Surface_ectoderm
120800000
120900000
121000000
121100000
chr8 position (bp)

Supplementary Figure 4. The tissue specific chromatin accessibility of *Foxf1* regulatory genomic regions in mouse embryos at E8.5.

The dataset was obtained using the accession number GSE133244 and further analyzed. Highlighted regions correspond to *Foxf1*-regulatory elements identified by our multiome dataset from E18.5 lungs. FEL2 is detected as accessible region in mesenchymal cells of mouse embryo at E8.5 (asterisk). FEL1, FEL2 and FEL4 are not accessible by Tn5 transposase in E8.5 embryos.

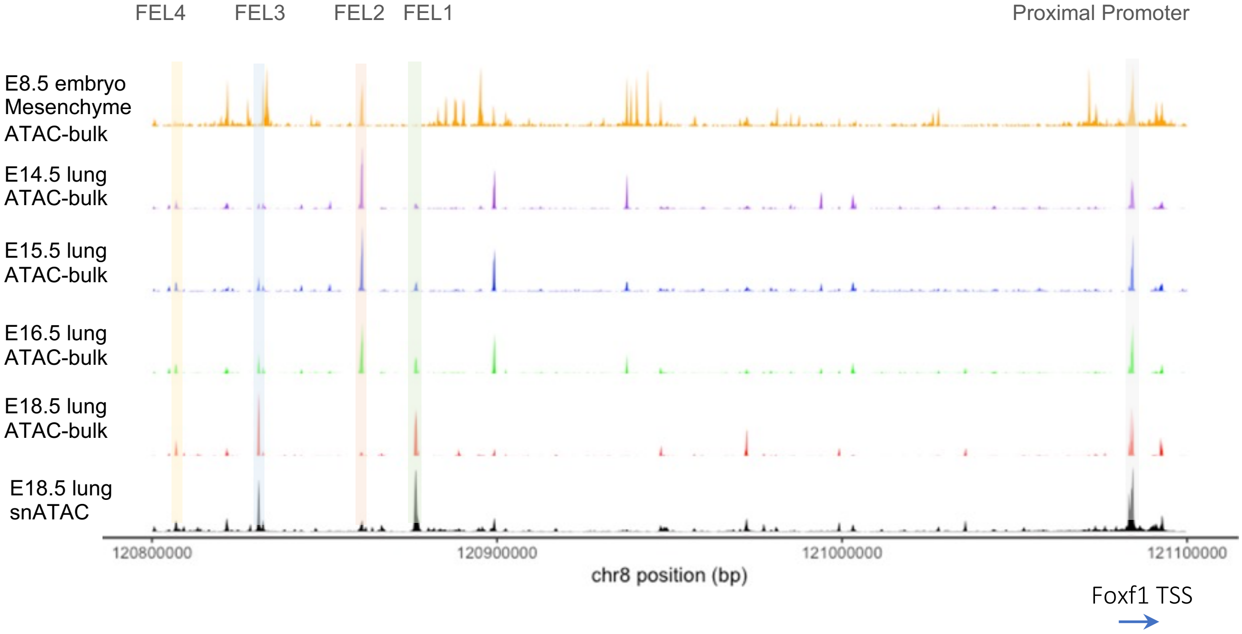

Supplementary Figure 5. The alignment of *Foxf1* distal regulatory elements to bulkATACseq datasets from several stages of mouse embryonic development.

The alignment of snATAC data from multiome E18.5 sequencing to the publicly available bulkATACseq obtained from different developmental stages: E8.5, E14.5, E15.5, E16.5 and E18.5. There are 4 shared accessible regions in *Foxf1* upstream sequences in addition to an accessible region in the *Foxf1* promoter. The FEL1, FEL3 and FEL4 are accessible at late developmental stages, whereas FEL2 is accessible at early developmental stages. TSS is transcriptional start site.

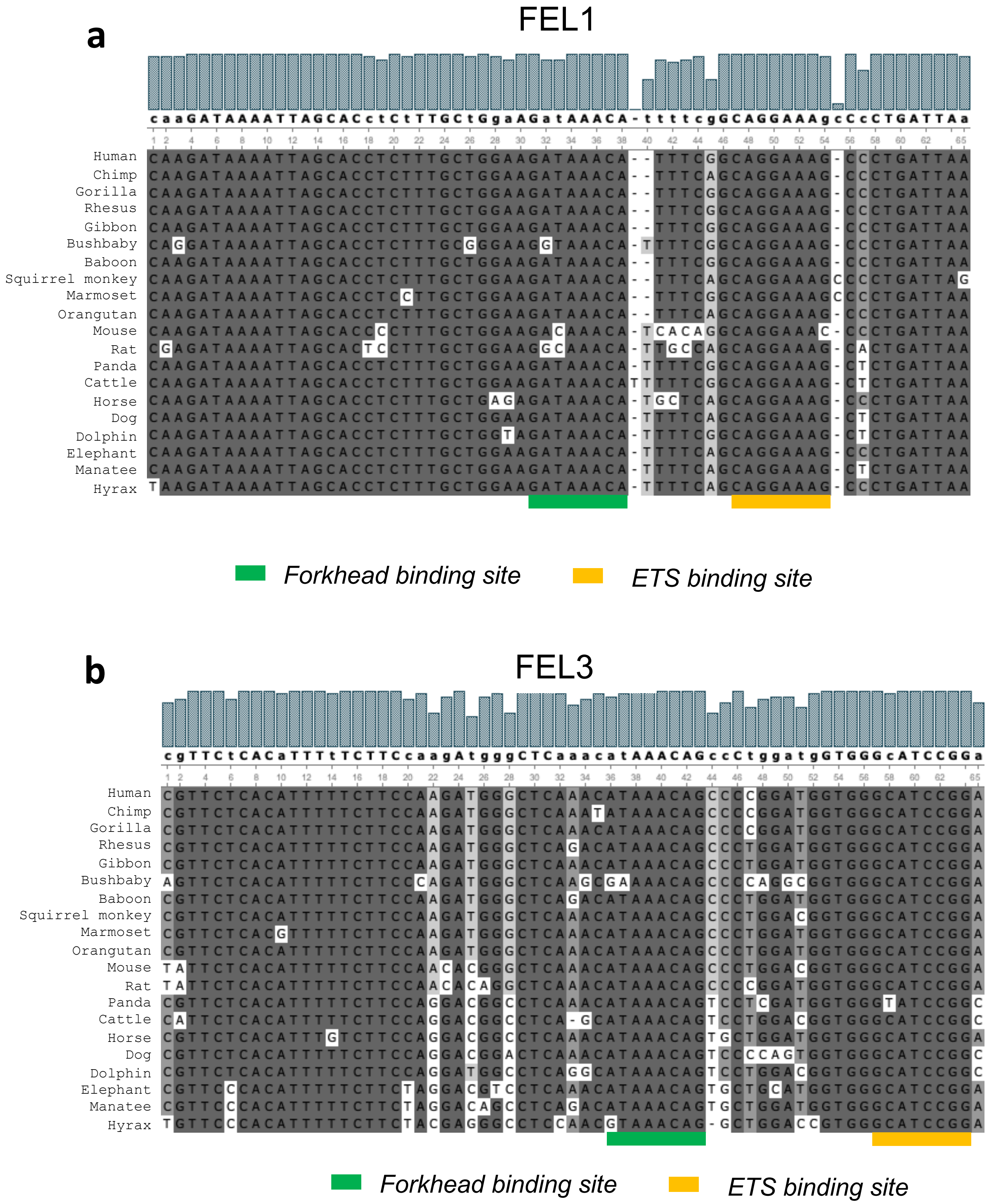
a
FEL1
caaGATAAAATTAGCACctCtTTGCtGgaAGatAAACA-ttttcgGCAGGAAAgcCcCTGATTAa
Human
Chimp
Gorilla
Rhesus
Gibbon
Bushbaby
Baboon
Squirrel monkey
Marmoset
Orangutan
Mouse
Rat
Panda
Cattle
Horse
Dog
Dolphin
Elephant
Manatee
Hyrax
Forkhead binding site
ETS binding site
b
FEL3
cgTTCtCACaTTTtTCTTCcaagAtgggCTCaaacatAAACAGccCtggatgGTGGGcATCCGGa
Human
Chimp
Gorilla
Rhesus
Gibbon
Bushbaby
Baboon
Squirrel monkey
Marmoset
Orangutan
Mouse
Rat
Panda
Cattle
Horse
Dog
Dolphin
Elephant
Manatee
Hyrax
Forkhead binding site
ETS binding site

Supplementary Figure 6. DNA sequences of distal regulatory enhancers FEL1 and FEL3.

DNA alignments of distal regulatory enhancers FEL1 (**a**) and FEL3 (**b**) were retrieved using UCSC genome browser. DNA sequences are aligned using ClustalW2 algorithm. The *Forkhead*-binding motif and ETS-binding motif are denoted by green and orange boxes, respectively.

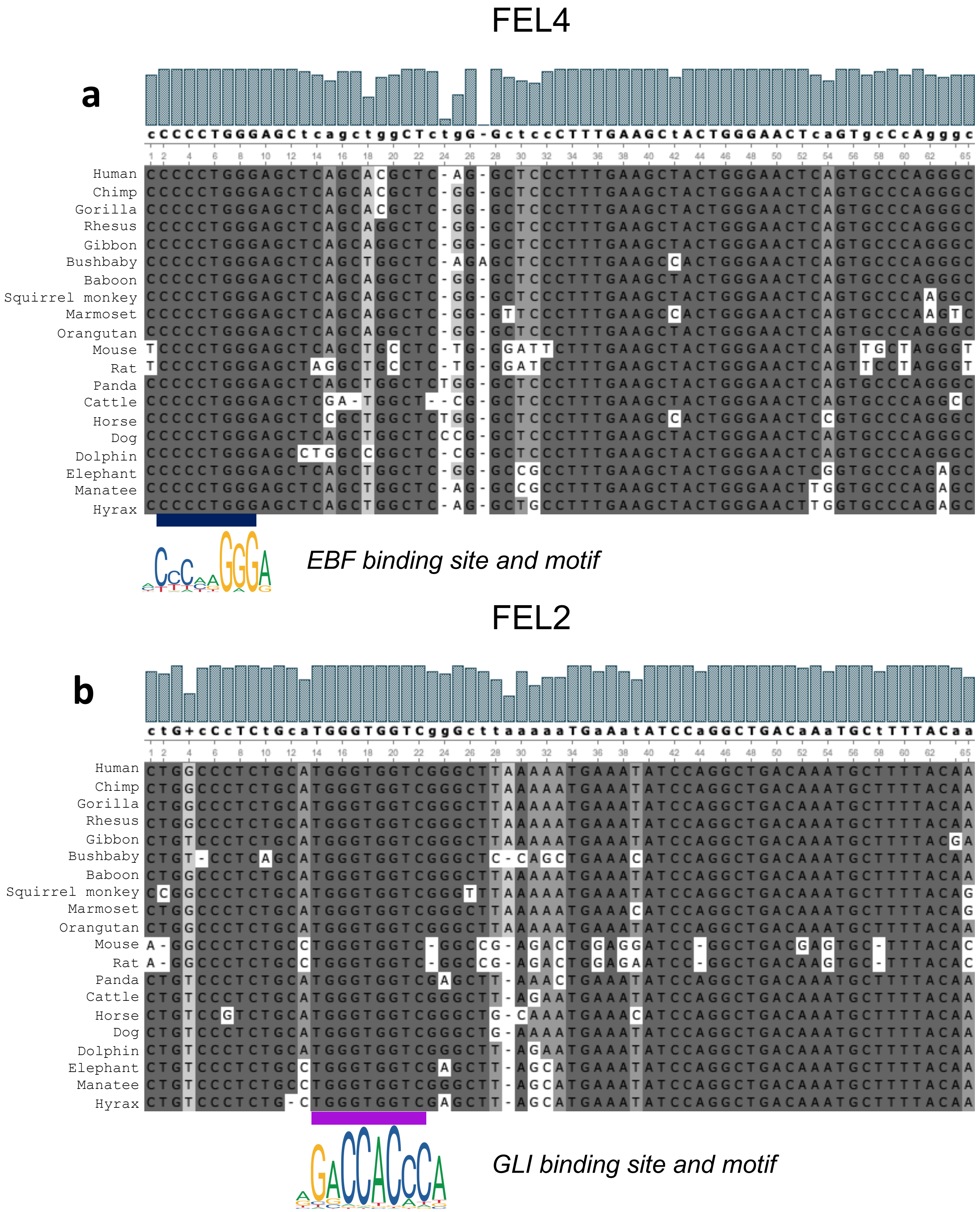
FEL4
a
Human
Chimp
Gorilla
Rhesus
Gibbon
Bushbaby
Baboon
Squirrel monkey
Marmoset
Orangutan
Mouse
Rat
Panda
Cattle
Horse
Dog
Dolphin
Elephant
Manatee
Hyrax
EBF binding site and motif
FEL2
b
Human
Chimp
Gorilla
Rhesus
Gibbon
Bushbaby
Baboon
Squirrel monkey
Marmoset
Orangutan
Mouse
Rat
Panda
Cattle
Horse
Dog
Dolphin
Elephant
Manatee
Hyrax
GLI binding site and motif

Supplementary Figure 7. DNA sequences of distal regulatory enhancers FEL4 and FEL2.

a, The sequence alignment of the distal regulatory element FEL4. The fasta sequences of the FEL4 enhancer in different species are downloaded using UCSC genome browser and the sequences were aligned using the ClustalW2 algorithm. The EBF-binding site is shown by the dark blue box. **b,** The sequence alignment of the distal regulatory element FEL2. The GLI-binding site is shown by the purple box.

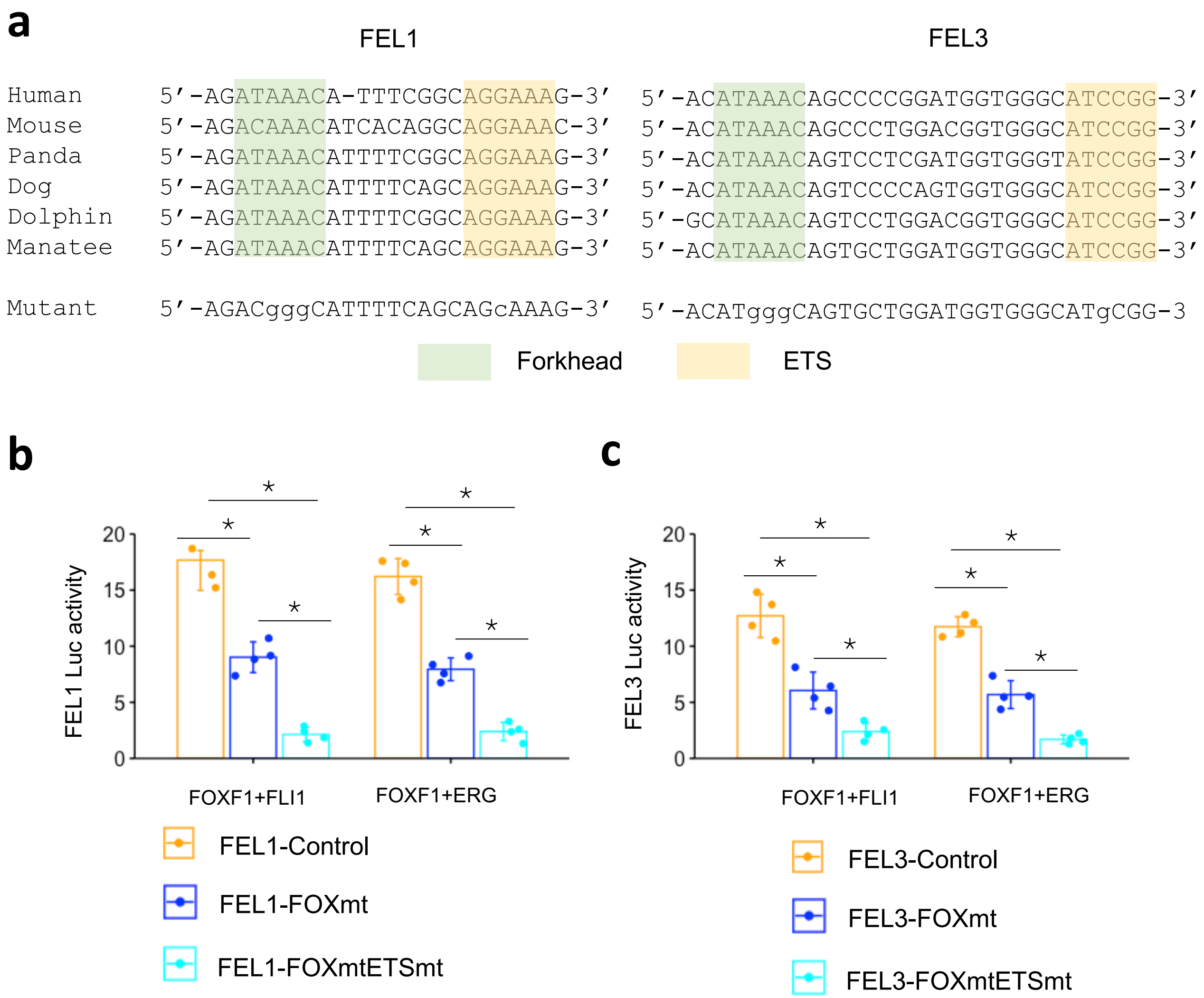

Supplementary Figure 8. The dual luciferase assay shows that the activity of FEL1 and FEL3 enhancers is dependent upon FOX- and ETS-binding motifs.

a, Schematic representation of mutations to disrupt FOX and ETS motifs in FEL1 and FEL3 enhancers. **b-c,** The dual luciferase assay shows that both FOX-binding site and ETS-binding site contribute to the enhancer activity in FEL1 and FEL3 regulatory regions. The *FOXF1* expression vector cooperates with either FLI1 or ERG expression vectors to activate FEL1 and FEL3, * is $p<0.05$. Source data are provided as a Source Data file.

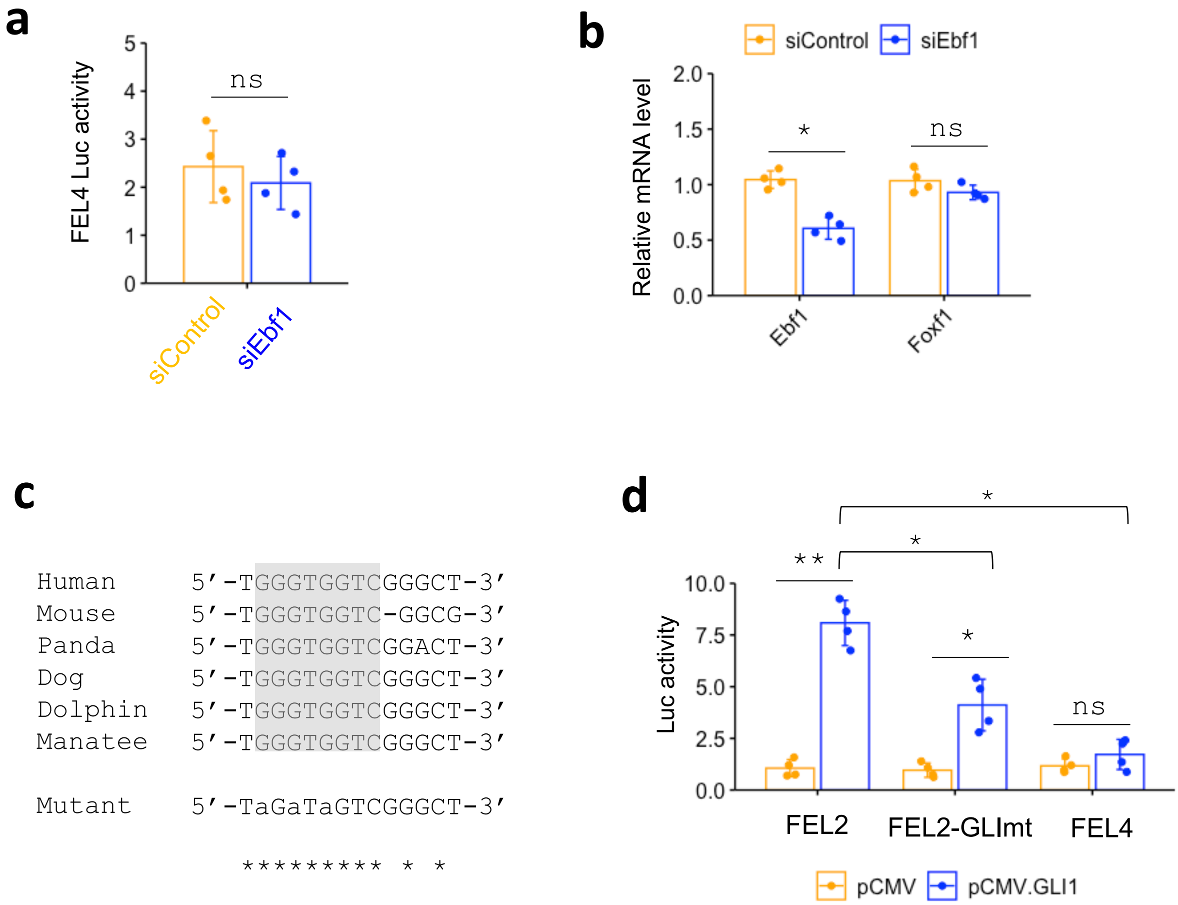

Supplementary Figure 9. Regulation of FEL2 and FEL4 enhancers by GLI1 and EBF1.

a, siRNA-mediated inhibition of *Ebf1* does not change the FEL4 activity. **b,** Real-time RT-PCR analysis shows that a 50% inhibition of *Ebf1* is not sufficient to reduce *Foxf1* mRNA. **c,** The diagram shows the mutation to disrupt the putative GLI-binding motifs in the FEL2 enhancer. **d,** The luciferase assay indicates that the GLI1 expression vector significantly increases FEL2 activity. FEL2 activity is dependent on the GLI-binding site. The activity of FEL4 is not changed after transfection with the GLI1 expression plasmid, * is $p<0.05$, ** is $p<0.01$, ns is not significant. Source data are provided as a Source Data file.

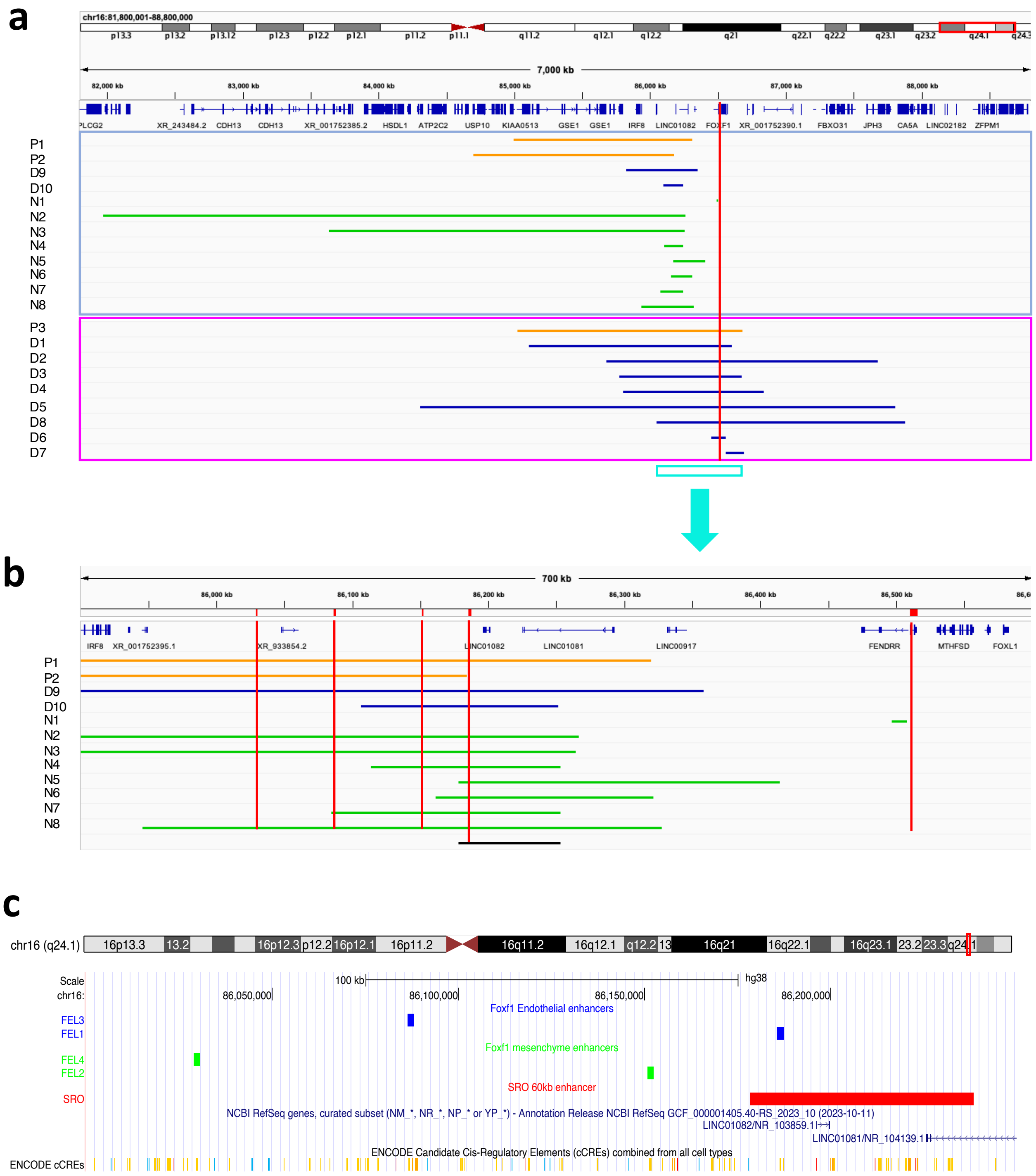
a
chr16:81,800,001-88,800,000
p13.3
p13.2
p13.12
p12.3
p12.2
p12.1
p11.2
p11.1
q11.2
q12.1
q12.2
q21
q22.1
q22.2
q23.1
q23.2
q24.1
7,000 kb
82,000 kb
83,000 kb
84,000 kb
85,000 kb
86,000 kb
87,000 kb
88,000 kb
PLCG2
XR_243484.2
CDH13
CDH13
XR_001752385.2
HSDL1
ATP2C2
USP10
KIAA0513
GSE1
GSE1
IRF8
LINC01082
FOXF1
XR_001752390.1
FBXO31
JPH3
CA5A
LINC02182
ZFPM1
P1
P2
D9
D10
N1
N2
N3
N4
N5
N6
N7
N8
P3
D1
D2
D3
D4
D5
D8
D6
D7
b
700 kb
86,000 kb
86,100 kb
86,200 kb
86,300 kb
86,400 kb
86,500 kb
IRF8
XR_001752395.1
XR_933854.2
LINC01082
LINC01081
LINC00917
FENDRR
MTHFSD
FOXL1
P1
P2
D9
D10
N1
N2
N3
N4
N5
N6
N7
N8
c
chr16 (q24.1)
16p13.3
13.2
16p12.3
p12.2
16p12.1
16p11.2
16q11.2
16q12.1
q12.2
13
16q21
16q22.1
16q23.1
23.2
23.3
q24
1
Scale
chr16:
100 kb
hg38
86,050,000
86,100,000
86,150,000
86,200,000
Foxf1 Endothelial enhancers
FEL3
FEL1
Foxf1 mesenchyme enhancers
FEL4
FEL2
SRO 60kb enhancer
SRO
NCBI RefSeq genes, curated subset (NM_*, NR_*, NP_* or YP_*) - Annotation Release NCBI RefSeq GCF_000001405.40-RS_2023_10 (2023-10-11)
LINC01082/NR_103859.1
LINC01081/NR_104139.1
ENCODE Candidate Cis-Regulatory Elements (cCREs) combined from all cell types
ENCODE cCREs

Supplementary Figure 10. The alignment of 16q24 genomic deletions in ACDMPV patients.

a, The alignment of 21 genomic 16q24 deletions in ACDMPV patients. Twelve genomic deletions do not affect the *FOXF1* coding sequence, 9 genomic deletions disrupt both *FOXF1* coding sequence and non-coding sequences. The vertical line marks the proximal *FOXF1* promoter. **b,** The alignment of *FOXF1* regulatory elements in 12 ACDMPV cases with non-coding genomic deletions. The previously reported 75kb shared deletion region (SDR) is shown by the black box. **c,** The diagram shows the relation of the previously characterized SRO (~60kb) regulatory region with newly identified FEL1, 2, 3 and 4 endothelial and mesenchymal enhancers. The FEL1 enhancer is shown as 2kb interval flanking the core sequence for better visualization. FEL2, 3, and 4 enhancers are shown as 1.5kb intervals. Endothelial enhancers (FEL1 and FEL3) are shown in blue. Mesenchymal enhancers (FEL2 and FEL4) are shown in green. The SRO region is shown in red. FEL1 is localized upstream of non-coding RNA LINC 01082 and overlaps with the SRO region.

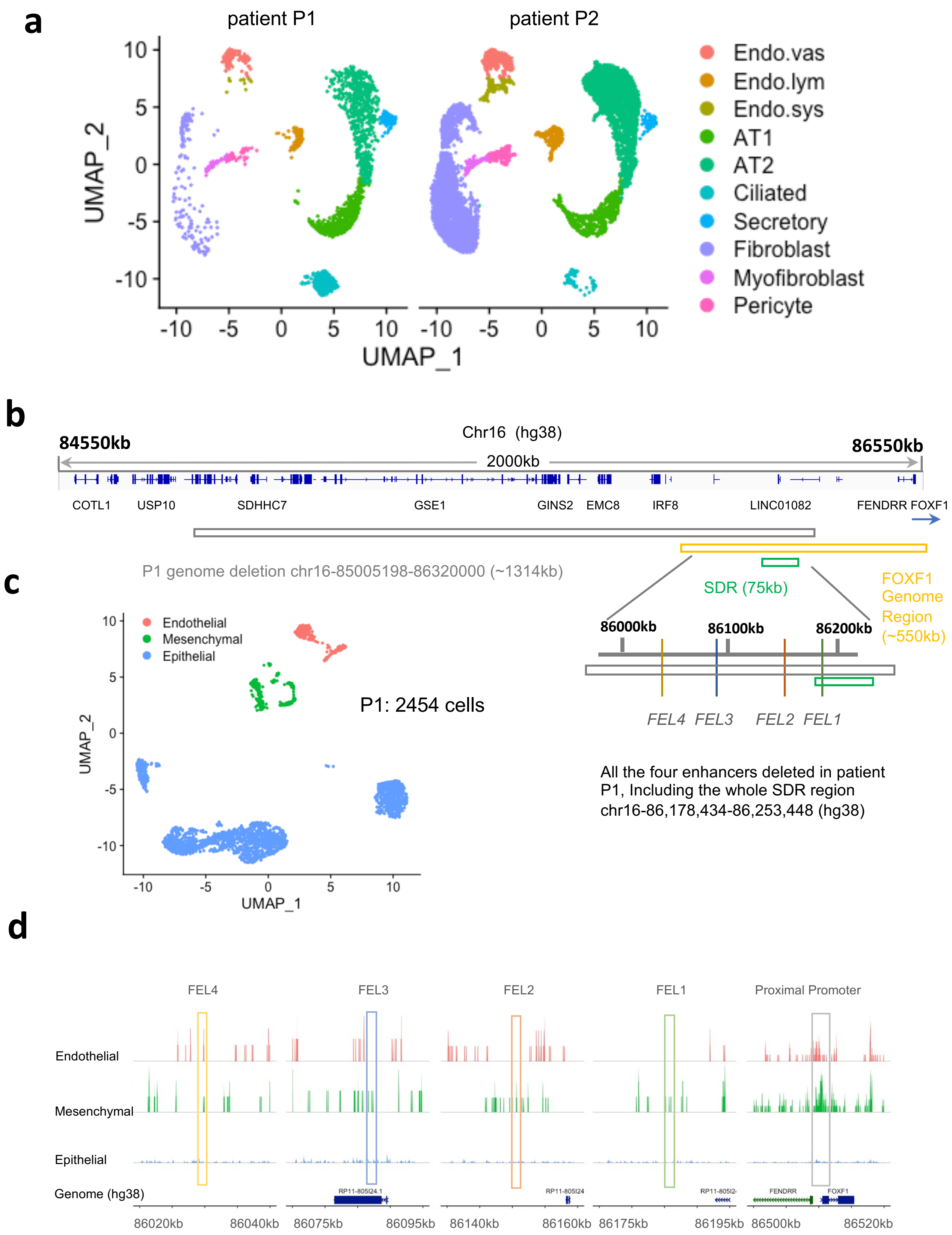
a
patient P1
patient P2
UMAP_2
UMAP_1
Endo.vas
Endo.lym
Endo.sys
AT1
AT2
Ciliated
Secretory
Fibroblast
Myofibroblast
Pericyte
b
Chr16 (hg38)
84550kb
86550kb
2000kb
COTL1
USP10
SDHHC7
GSE1
GINS2
EMC8
IRF8
LINC01082
FENDRR FOXF1
P1 genome deletion chr16-85005198-86320000 (~1314kb)
SDR (75kb)
FOXF1 Genome Region (~550kb)
c
Endothelial
Mesenchymal
Epithelial
P1: 2454 cells
86000kb
86100kb
86200kb
FEL4 FEL3 FEL2 FEL1
All the four enhancers deleted in patient P1, Including the whole SDR region chr16-86,178,434-86,253,448 (hg38)
d
FEL4
FEL3
FEL2
FEL1
Proximal Promoter
Endothelial
Mesenchymal
Epithelial
Genome (hg38)
FENDRR
FOXF1
86020kb
86040kb
86075kb
86095kb
86140kb
86160kb
86175kb
86195kb
86500kb
86520kb

Supplementary Figure 11. The multiome analysis of lung samples from ACDMPV patients.

a, The integrative UMAP projection of GEX libraries from 10X multiome sequencing of ACDMPV patient P1 and patient P2. Similar cell sub-clusters are seen in both datasets. **b,** Schematic diagram shows the genomic deletion (grey) in P1 ACDMPV patient in the context of 16q24.1 region. The *FOXF1* genomic region is highlighted by the orange box. The green box indicates the 75kb shared deletion region (SDR). The illustration shows an overlap of the *FOXF1* genomic deletion in patient P2 which includes FEL2-4 but not FEL1.The annotated coordinates are derived from the human genome assembly GRCh38. **c,** The UMAP joint projection and unsupervised clustering of non-hematopoietic lung cells (corresponding to CD45-negative cells in flow cytometry). **d,** The genome alignment of *FOXF1* regulatory elements is performed using single-nuclei ATACseq from the multiome sequencing from patient P1. The accessibility of the *FOXF1* proximal promoter region is detected, but the accessibility of endothelial regulatory elements FEL1 and FEL3 is undetectable. The alignment of *FOXF1* regulatory elements is performed using single-nuclei multiome sequencing from healthy donor lung. The 20kb adjacent regions are shown for each regulatory element.

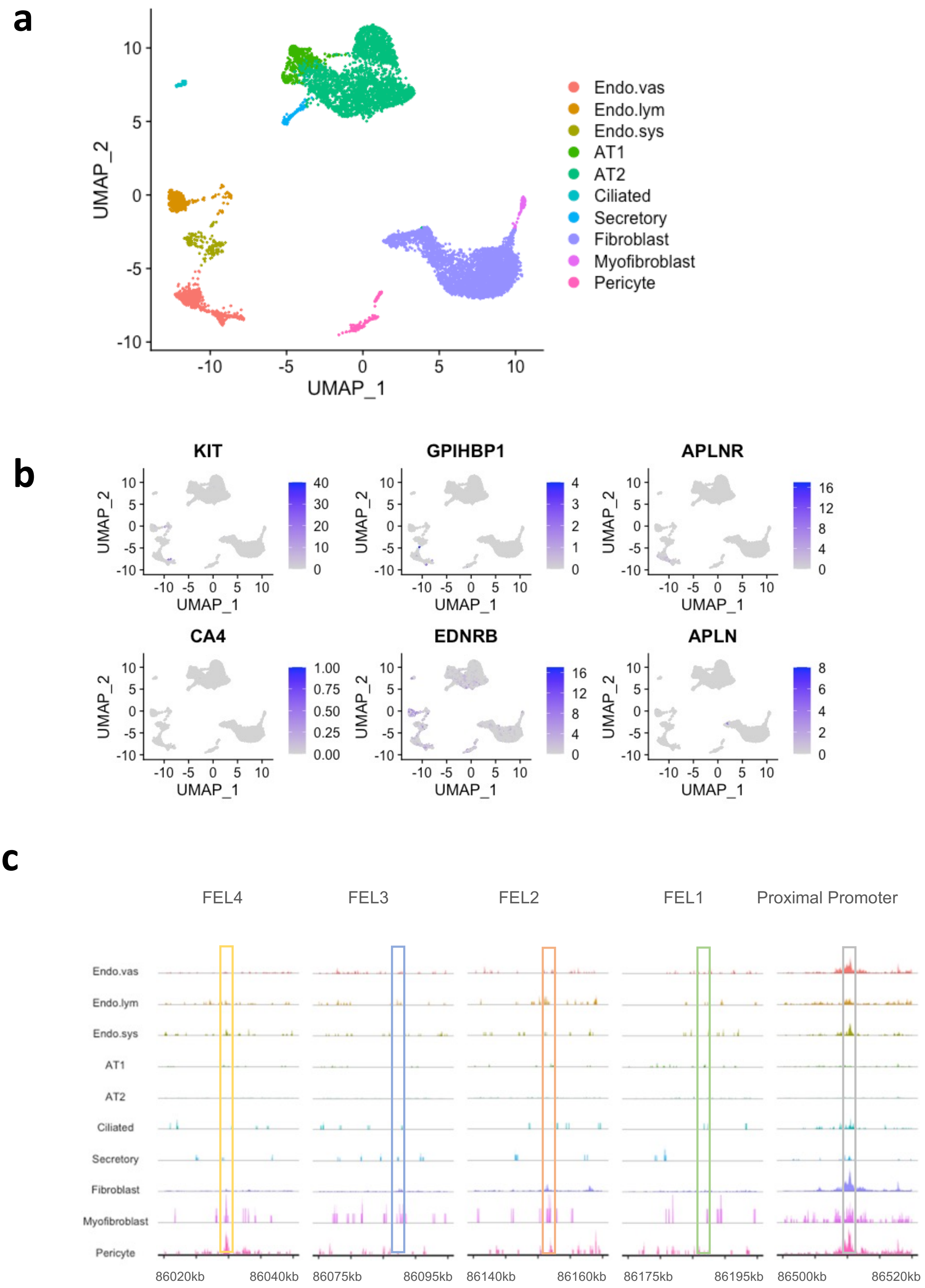
a
UMAP_2
UMAP_1
Endo.vas
Endo.lym
Endo.sys
AT1
AT2
Ciliated
Secretory
Fibroblast
Myofibroblast
Pericyte
b
KIT
GPIHBP1
APLNR
CA4
EDNRB
APLN
c
FEL4
FEL3
FEL2
FEL1
Proximal Promoter
86020kb
86040kb
86075kb
86095kb
86140kb
86160kb
86175kb
86195kb
86500kb
86520kb

Supplementary Figure 12. The multiome analysis of ACDMPV lung tissue from patient P2.

a, The joint clustering of ACDMPV patient P2 multiome (GEX + ATAC) data. **b,** The featureplots of *CA4*, *EDNRB*, *APLN* (all are aCap markers) and KIT, GPIHBP1, APLNR (all are gCAP markers) show the absence of aCAP and gCAP endothelial cells in the ACDMPV dataset. **c,** The human *FOXF1* genome region corresponding to mouse *Foxf1* regulatory elements is identified using comparative genomic analysis from UCSC genome browser. For each region, 20kb sequences flanking the core regulatory region are shown.

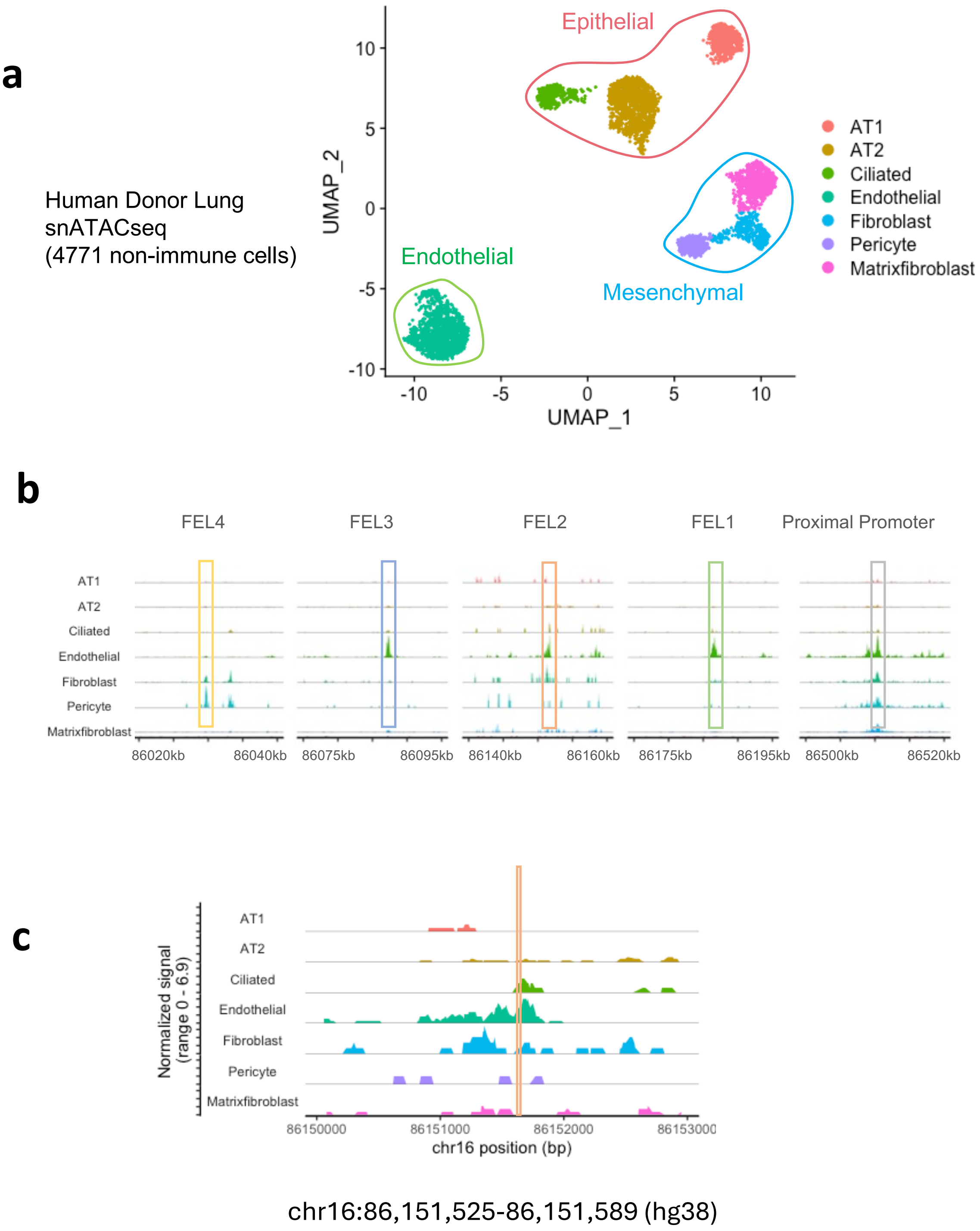
a
Human Donor Lung
snATACseq
(4771 non-immune cells)
Epithelial
Endothelial
Mesenchymal
UMAP_1
UMAP_2
10
5
0
-5
-10
AT1
AT2
Ciliated
Endothelial
Fibroblast
Pericyte
Matrixfibroblast
b
FEL4
FEL3
FEL2
FEL1
Proximal Promoter
AT1
AT2
Ciliated
Endothelial
Fibroblast
Pericyte
Matrixfibroblast
86020kb
86040kb
86075kb
86095kb
86140kb
86160kb
86175kb
86195kb
86500kb
86520kb
c
Normalized signal
(range 0 - 6.9)
AT1
AT2
Ciliated
Endothelial
Fibroblast
Pericyte
Matrixfibroblast
86150000
86151000
86152000
86153000
chr16 position (bp)
chr16:86,151,525-86,151,589 (hg38)

Supplementary Fig 13. The analysis of single nuclei ATACseq dataset from healthy human donor lung.

a, Clustering of non-hematopoietic cells in the snATACseq dataset from healthy human donor lung. The annotation of cell clusters is adapted from GSE161383 dataset. **b,** Human *FOXF1* genome regulatory elements corresponding to mouse *Foxf1* regulatory elements are identified based on the comparative genomic analysis. For each region, 20kb sequences flanking the core regulatory region are shown. **c,** Schematic shows the FEL2 enhancer, the size of which is ~400bp based on evolutionary conservation. The narrow peak harboring the potential GLI-binding site (65bp, orange box) is located within a region of increased chromatin accessibility in both pulmonary endothelial cells and lung fibroblasts.

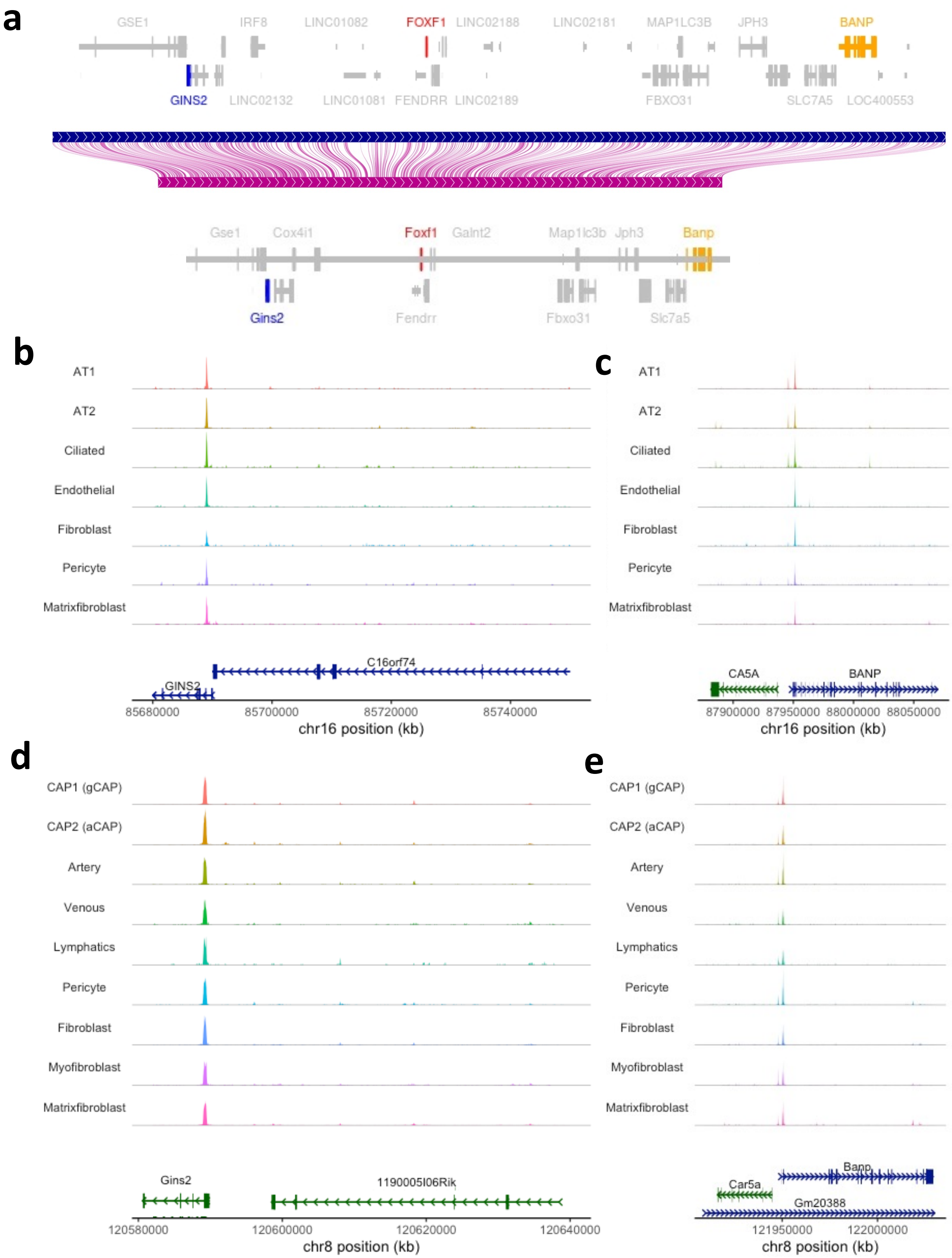

a
GSE1
IRF8
LINC01082
FOXF1
LINC02188
LINC02181
MAP1LC3B
JPH3
BANP
GINS2
LINC02132
LINC01081
FENDRR
LINC02189
FBXO31
SLC7A5
LOC400553
Gse1
Cox4i1
Foxf1
Galnt2
Map1lc3b
Jph3
Banp
Gins2
Fendrr
Fbxo31
Slc7a5
b
AT1
AT2
Ciliated
Endothelial
Fibroblast
Pericyte
Matrixfibroblast
C16orf74
GINS2
85680000
85700000
85720000
85740000
chr16 position (kb)
c
AT1
AT2
Ciliated
Endothelial
Fibroblast
Pericyte
Matrixfibroblast
CA5A
BANP
87900000
87950000
88000000
88050000
chr16 position (kb)
d
CAP1 (gCAP)
CAP2 (aCAP)
Artery
Venous
Lymphatics
Pericyte
Fibroblast
Myofibroblast
Matrixfibroblast
Gins2
1190005I06Rik
120580000
120600000
120620000
120640000
chr8 position (kb)
e
CAP1 (gCAP)
CAP2 (aCAP)
Artery
Venous
Lymphatics
Pericyte
Fibroblast
Myofibroblast
Matrixfibroblast
Banp
Car5a
Gm20388
121950000
122000000
chr8 position (kb)

Supplementary Figure 14. The comparative analysis shows human and mouse genomic regions in the proximity of the *FOXF1* gene based on synteny of chromosomes.

a, The diagram of the syntenic region flanking *FOXF1* in human (top) and *Foxf1 in* mouse genomes (bottom). The genome organization is highly conserved upstream and downstream of *Foxf1* mouse and *FOXF1* human genes. **b-e,** *Gins2* and *Banp* are two genes in the proximity to *Foxf1* in both mouse and human. The open chromatin profile in *Gins2* promoter is similar between human (**b)** and mouse genomes (**d)**. The chromatin accessibility in the *Banp* promoter is similar between human (**c)** and mouse genomes (**e)**.

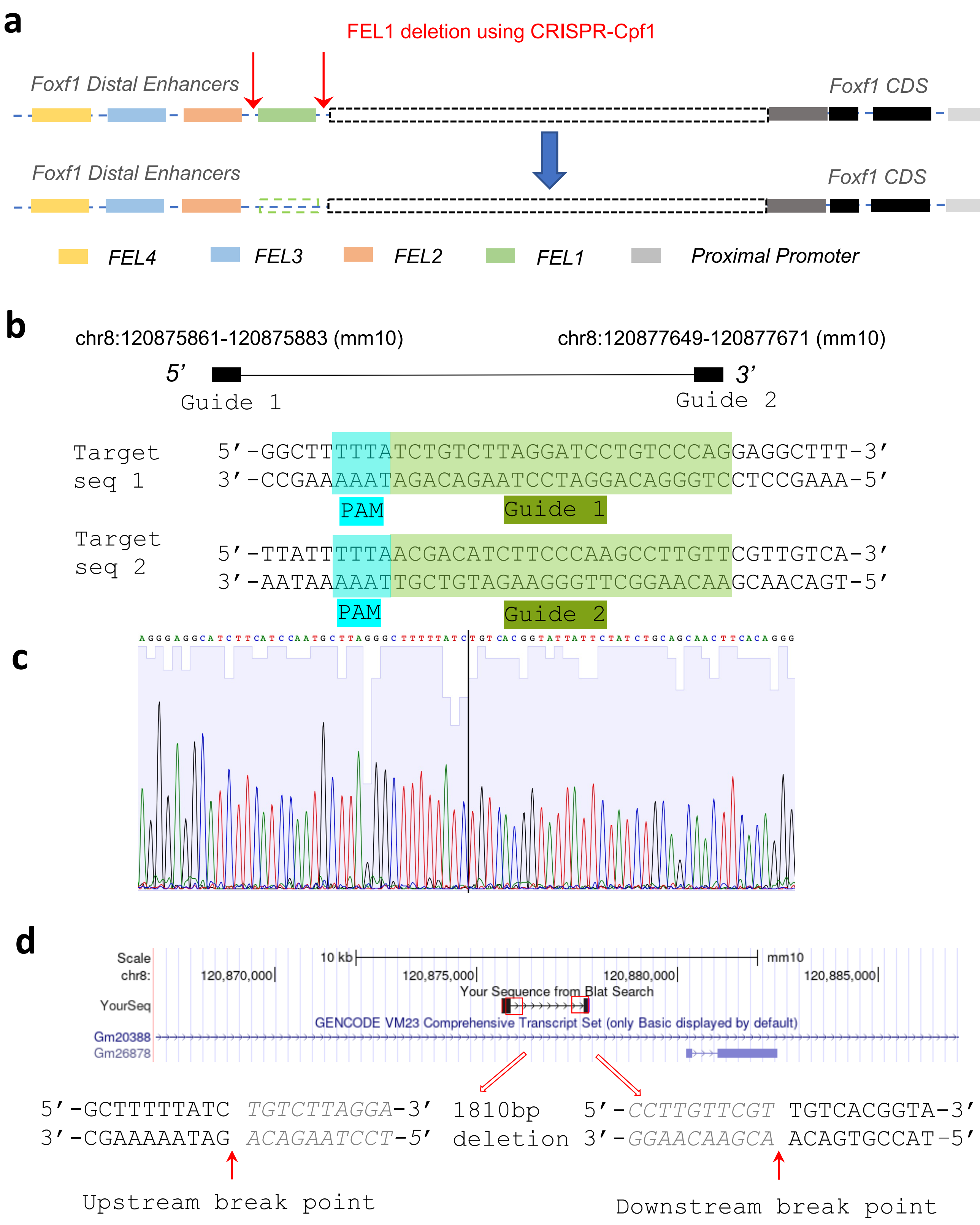
a
FEL1 deletion using CRISPR-Cpf1
Foxf1 Distal Enhancers
Foxf1 CDS
Foxf1 Distal Enhancers
Foxf1 CDS
FEL4
FEL3
FEL2
FEL1
Proximal Promoter
b
chr8:120875861-120875883 (mm10)
chr8:120877649-120877671 (mm10)
5'
3'
Guide 1
Guide 2
Target seq 1
5'-GGCTTTTTATCTGTCTTAGGATCCTGTCCCAGGAGGCTTT-3'
3'-CCGAAAAATAGACAGAATCCTAGGACAGGGTCCTCCGAAA-5'
PAM
Guide 1
Target seq 2
5'-TTATTTTTAACGACATCTTCCCAAGCCTTGTTCGTTGTCA-3'
3'-AATAAAAATTGCTGTAGAAGGGTTCGGAACAAGCAACAGT-5'
PAM
Guide 2
c
d
Scale
10 kb
mm10
chr8:
120,870,000
120,875,000
120,880,000
120,885,000
Your Sequence from Blat Search
YourSeq
GENCODE VM23 Comprehensive Transcript Set (only Basic displayed by default)
Gm20388
Gm26878
5'-GCTTTTTATC TGTCTTAGGA-3'
3'-CGAAAAATAG ACAGAATCCT-5'
1810bp deletion
5'-CCTTGTTCGT TGTCACGGTA-3'
3'-GGAACAAGCA ACAGTGCCAT-5'
Upstream break point
Downstream break point

Supplementary Figure 15. The multiplex genome editing of FEL1 using CRISPR-Cpf1.

a, The schematic diagram shows the strategy to disrupt the FEL1 enhancer using the multiplex genome editing with CRISPR-Cpf1. **b,** Sequences show guide RNAs used to disrupt the FEL1 regulatory element. **c**, The chromatogram of Sanger DNA sequencing of the PCR fragment flanking FEL1 genomic fragment. Black line shows the genome break point after CRISPR genome editing. **d**, The detailed sequence of the upstream and downstream break point in genomic DNA from mutant ESCs.

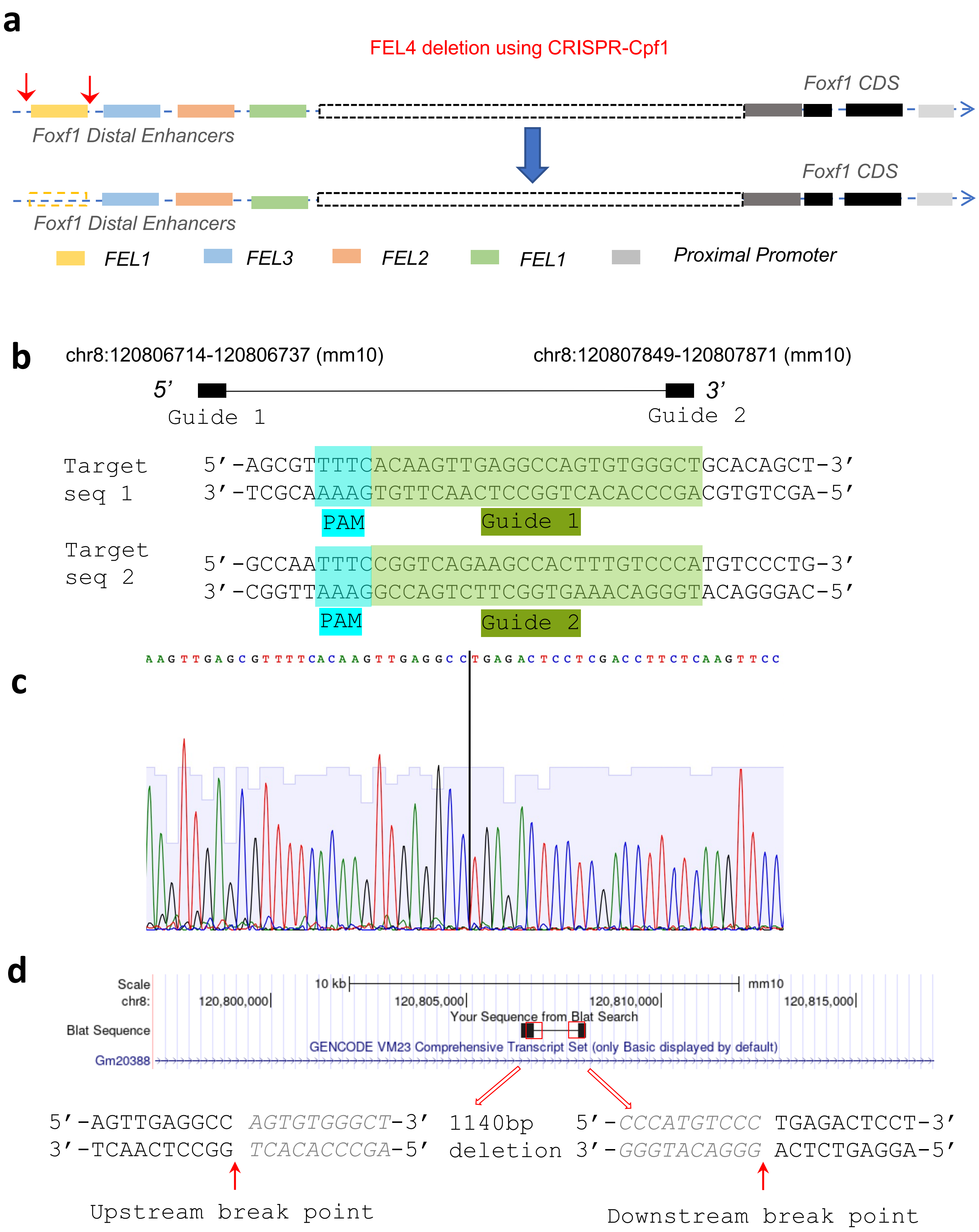
a
FEL4 deletion using CRISPR-Cpf1
Foxf1 CDS
Foxf1 Distal Enhancers
Foxf1 CDS
Foxf1 Distal Enhancers
FEL1
FEL3
FEL2
FEL1
Proximal Promoter
b
chr8:120806714-120806737 (mm10)
chr8:120807849-120807871 (mm10)
5’
3’
Guide 1
Guide 2
Target seq 1
5’-AGCGTTTTCACAAGTTGAGGCCAGTGTGGGCTGCACAGCT-3’
3’-TCGCAAAAGTGTTCAACTCCGGTCACACCCGACGTGTCGA-5’
PAM
Guide 1
Target seq 2
5’-GCCAATTTCCGGTCAGAAGCCACTTTGTCCCATGTCCCTG-3’
3’-CGGTTAAAGGCCAGTCTTCGGTGAAACAGGGTACAGGGAC-5’
PAM
Guide 2
c
AAGTTGAGCGTTTTCACAAGTTGAGGCCTGAGACTCCTCGACCTTCTCAAGTTCC
d
Scale
chr8:
10 kb
mm10
120,800,000
120,805,000
120,810,000
120,815,000
Your Sequence from Blat Search
Blat Sequence
GENCODE VM23 Comprehensive Transcript Set (only Basic displayed by default)
Gm20388
5’-AGTTGAGGCC AGTGTGGGCT-3’
3’-TCAACTCCGG TCACACCCGA-5’
1140bp deletion
5’-CCCATGTCCC TGAGACTCCT-3’
3’-GGGTACAGGG ACTCTGAGGA-5’
Upstream break point
Downstream break point

Supplementary Figure 16. The multiplex genome editing of FEL4 using CRISPR-Cpf1.

a, The schematic diagram shows the strategy to disrupt the FEL4 enhancer using the multiplex genome editing with CRISPR-Cpf1. **b,** Sequences show guide RNAs used to disrupt the FEL4 regulatory element. **c,** The chromatogram of Sanger DNA sequencing of the PCR fragment flanking FEL4 genomic fragment. Black line shows the genome break point after CRISPR genome editing. **d**, The detailed sequence of the upstream and downstream break point in genomic DNA from mutant ESCs.

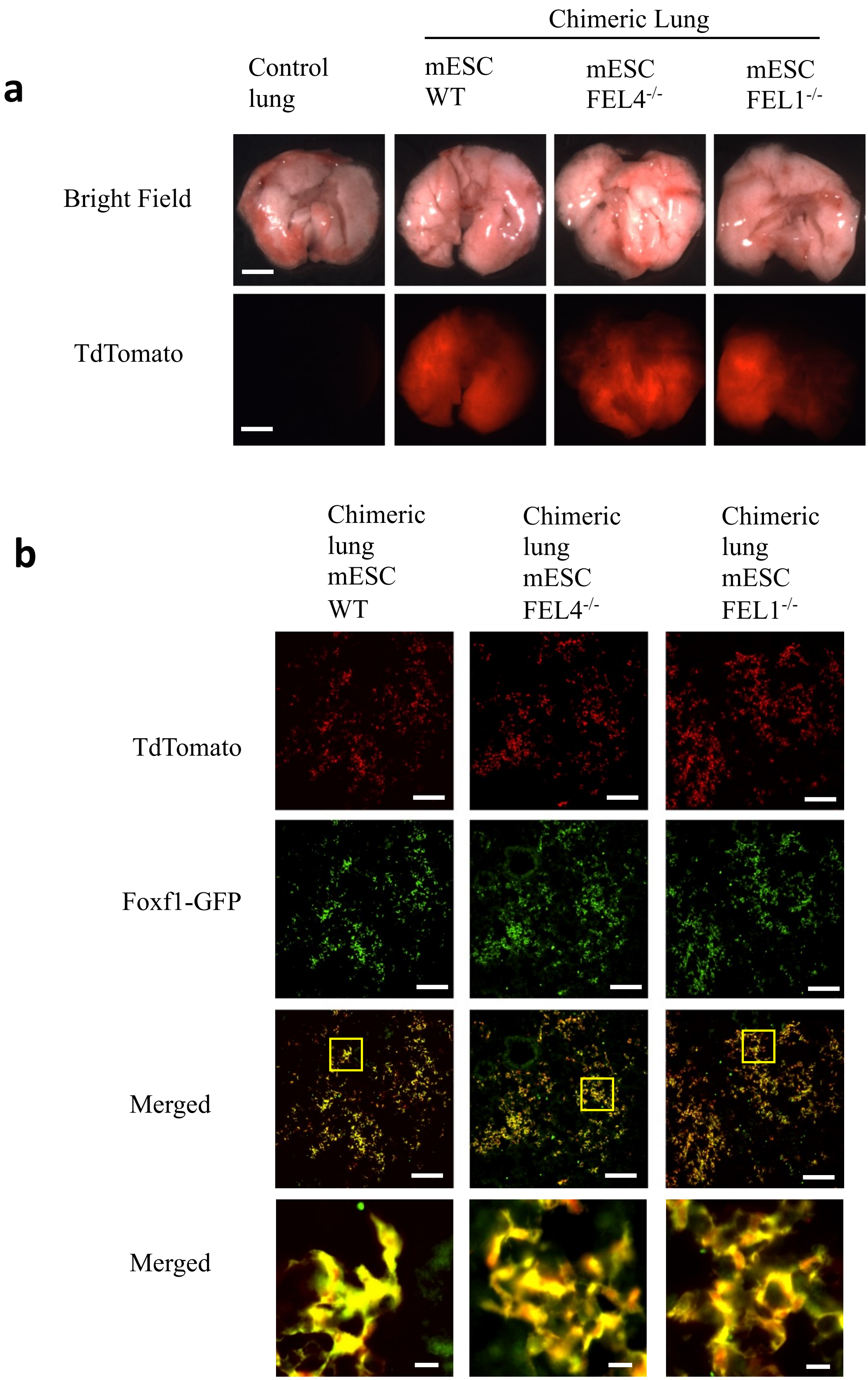
a
Chimeric Lung
Control lung
mESC WT
mESC FEL4-/-
mESC FEL1-/-
Bright Field
TdTomato
b
Chimeric lung mESC WT
Chimeric lung mESC FEL4-/-
Chimeric lung mESC FEL1-/-
TdTomato
Foxf1-GFP
Merged
Merged

Supplementary Figure 17. The functional validation of FEL1 and FEL4 enhancers using blastocyst complementation.

a, Images show chimeras produced by blastocyst complementation using wild type mESC (WT), and mutant FEL4$^{-/-}$ and FEL1$^{-/-}$ mESCs. Scale bars are 1mm. **b,** Images show tdTomato and GFP fluorescence from donor mESCs in chimeric lungs. Disruption of either FEL1 or FEL4 distal regulatory element does not affect the integration of mESCs into chimeric embryos. Scale bars are 100μm in top and middle images, and 10μm in bottom images.

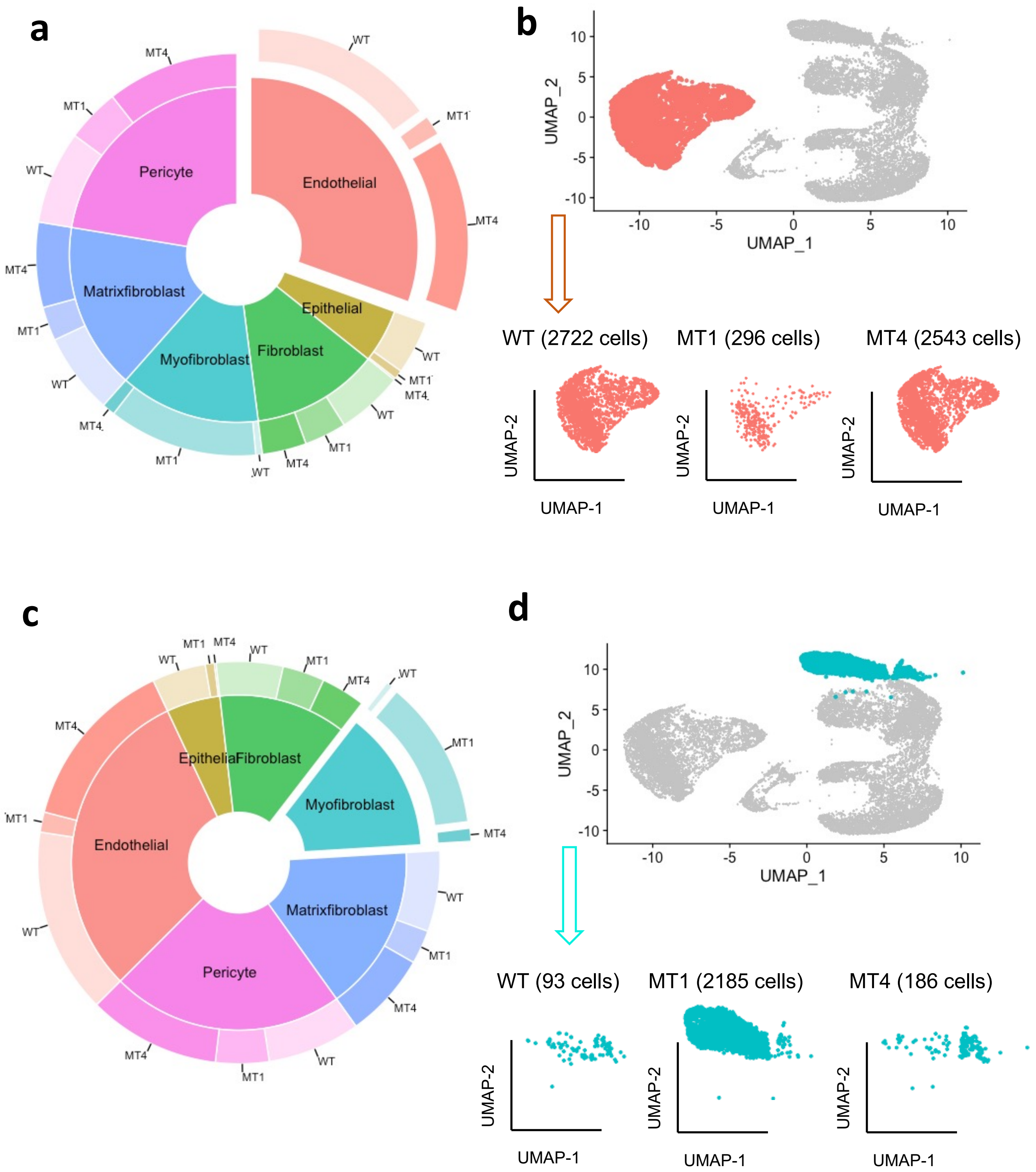

a
Endothelial
Pericyte
Matrixfibroblast
Myofibroblast
Fibroblast
Epithelial
WT
MT1
MT4
b
UMAP_2
UMAP_1
WT (2722 cells)
MT1 (296 cells)
MT4 (2543 cells)
UMAP-2
UMAP-1
c
Endothelial
EpitheliaFibroblast
Myofibroblast
Matrixfibroblast
Pericyte
d
WT (93 cells)
MT1 (2185 cells)
MT4 (186 cells)

Supplementary Figure 18. Single-cell RNA sequencing analysis shows that FEL1 deletion impairs endothelial differentiation and leads to aberrant myofibroblast accumulation.

a, Diagram shows ratios of cells from different subtypes identified by single-cell RNAseq analysis of tdTomato+ chimeric lung cells derived from control mESC s(WT), FEL1$^{-/-}$ mESCs (MT1), and FEL4$^{-/-}$ mESCs (MT4) via blastocyst complementation. Endothelial cells are reduced in MT1 cells. **b,** Featureplots show cellular distributions of endothelial cells in WT, MT1 and MT4 single-cell RNAseq datasets. **c,** Ratio of myofibroblasts is increased in MT1 cells. **d,** Featureplots show cellular distributions of myofibroblasts in WT, MT1 and MT4 datasets.

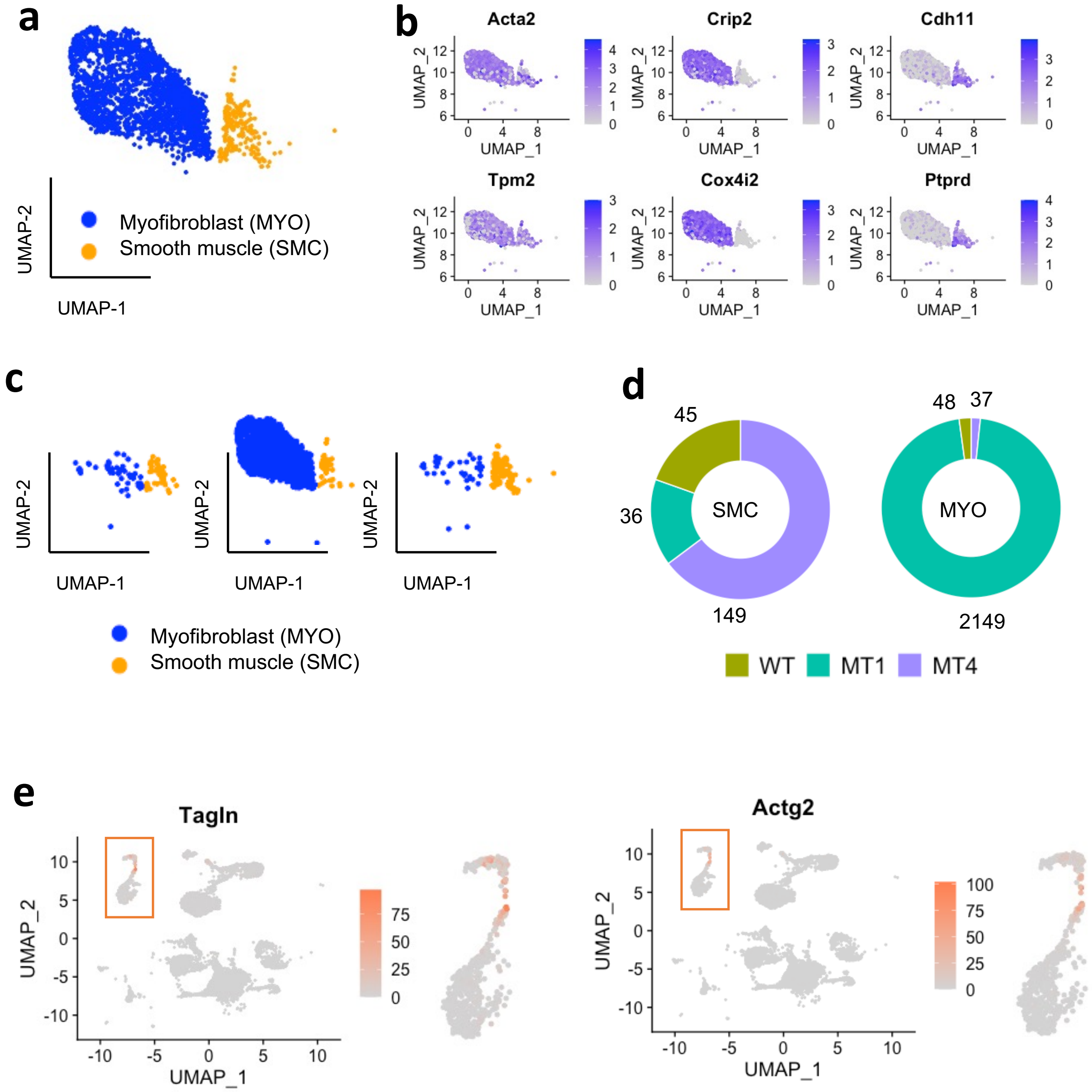
a
UMAP-2
UMAP-1
Myofibroblast (MYO)
Smooth muscle (SMC)
b
Acta2
Crip2
Cdh11
Tpm2
Cox4i2
Ptprd
UMAP_2
UMAP_1
c
UMAP-2
UMAP-1
Myofibroblast (MYO)
Smooth muscle (SMC)
d
45
36
149
SMC
48
37
MYO
2149
WT
MT1
MT4
e
Tagln
Actg2
UMAP_2
UMAP_1

Supplementary Figure 19. Single-cell RNA sequencing analysis shows that FEL4 deletion leads to aberrant accumulation of smooth muscle cells.

a-c, Featureplots show sub-clustering of myofibroblasts and smooth muscle cells. Single-cell RNAseq analysis was performed using tdTomato+ chimeric lung cells derived from control mESC s(WT), FEL1$^{-/-}$ mESCs (MT1), and FEL4$^{-/-}$ mESCs (MT4) via blastocyst complementation. Both myofibroblasts (MYO) and smooth muscle cells (SMC) express *Acta2* and *Tpm2*. Myofibroblasts selectively express *Cox4i2* and *Crip2*, whereas *Cdh11* and *Ptprd* mRNAs are highly enriched in smooth muscle cells. **c,** In MT1 sample with FEL1 deletion, myofibroblast significantly increased. **d,** Pieplots show cellular distributions of myofibroblasts and smooth muscle cells among WT, MT1 and MT4 single-cell RNAseq datasets. **e,** Featureplots show expression *Actg2* and *Tagln* in the population of smooth muscle cells from the multiome sequencing.

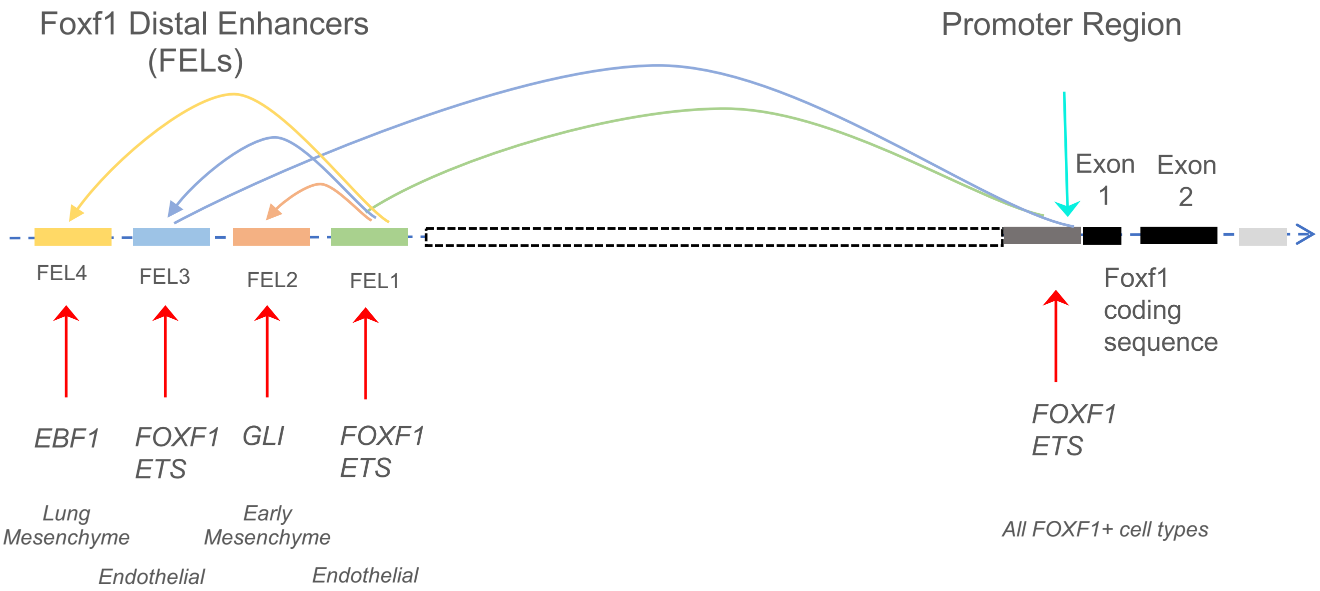

Supplementary Figure 20. The schematic diagram of the regulation of *Foxf1* gene expression by FEL regulatory elements.

The schematic diagram shows the transcriptional regulation of *Foxf1* expression in the lung. *Foxf1* expression is regulated by the cooperation of four distal FEL regulatory elements and the proximal *Foxf1* promoter region. FEL1 is active in lung endothelial cells, and it is required for *Foxf1* expression in both endothelial and mesenchymal cell lineages. FEL2 is active in the early embryonic development prior to the lung morphogenesis. FEL3 is primarily active in pulmonary endothelial cells, whereas FEL4 is a mesenchyme-specific regulatory element. The Foxf1 proximal promoter region is required for basal Foxf1 expression is all Foxf1-expressing cell types. The regulation of FEL enhancers by various transcription factors is show by red arrows.

Supplementary Table 1. Genomic deletions in 12 ACDMPV cases upstream of the FOXF1 coding sequence.

Patient	Gender	Survival Time	Genomic Deletion Range (hg38)	Deletion Size (bp)	FEL4	FEL3	FEL2	FEL1	PROMOTER	PMID
P1	M	35days	chr16-85005198-86320000	1,314,802	×	×	×	×	√	37463497
P2	M	30days	chr16-84704841-86184669	1,479,828	×	×	×	√	√	37463497
D9	F	20days	chr16-85834162-86358555	524,393	×	×	×	×	√	19500772
D10	F	13days	chr16-86106894-86251893	144,999	√	√	×	×	√	19500772
N1	NA	NA	chr16-86496893-86507893	11,000	√	√	√	√	×	23034409
N2	NA	NA	chr16-81981033-86266876	4,285,843	×	×	×	×	√	23034409
N3	NA	NA	chr16-83639776-86264773	2,624,997	×	×	×	×	√	23034409
N4	NA	NA	chr16-86113920-86253554	139,634	√	√	×	×	√	23034409
N5	NA	NA	chr16-86178434-86414553	236,119	√	√	√	×	√	23034409
N6	NA	NA	chr16-86161366-86321555	160,189	√	√	√	×	√	23034409
N7	NA	NA	chr16-86084524-86253459	168,935	√	×	×	×	√	23034409
N8	NA	NA	chr16-85945881-86327617	381,736	×	×	×	×	√	23034409

×: deleted √: unaffected

Supplementary Table 2. Primers used in the genomic engineering of FEL1 and FEL4 by CRISPR-Cpf1

sgFEL1-F	AGATTCTGTCTTAGGATCCTGTCCCAGAATTTCTACTAAGTGTAGATACGACATCTTCCCAAGCCTTGTT
sgFEL1-R	AAAAAACAAGGCTTGGGAAGATGTCGTATCTACACTTAGTAGAAATTCTGGGACAGGATCCTAAGACAGA
sgFEL4-F	AGATACAAGTTGAGGCCAGTGTGGGCTAATTTCTACTAAGTGTAGATCGGTCAGAAGCCACTTTGTCCCA
sgFEL4-R	AAAATGGGACAAAGTGGCTTCTGACCGATCTACACTTAGTAGAAATTAGCCCACACTGGCCTCAACTTGT

Supplementary Table 3. Primers used in the cloning and mutation

Primer name	Oilgo sequence
FEL1~F'	CCAGGTACCGGCATTAGCGATGGATCATTCTGACTAG
FEL1~R'	CAGGCTAGCGAGCTCACTGCCTATGTCAGACCAATACTATTTC
FEL2~F'	ACTGGTACCCACTGGGAGGTTCACGTGGAGATGC
FEL2~R'	GAGGCTAGCTGACTGCAGCAGAGCAGGCTGCCTCAC
FEL3~F'	CCTGGTACCCAGCGACCTTACCCAGCCACCATGTTC
FEL3~R'	TCTGCTAGCGAGCTCCAGTGGCAGACAGGGACAGCGGGGTG
FEL4~F'	CTGGGTACCTTGCAGGCAGCTCCGGGGACTTTGTTC
FEL4~R'	AATGCTAGCCATTACCCTGAGAAACACACTGCTACG
Ebf1F	GCTGAATTCATGTTTGGGATCCAGGAAAGCATC
Ebf1R	ATCTCTAGATCACATGGGAGGGACAATCATGCC
FEL1m~F'	GCACCCCTTTGCTGGAAGACGGGCATCACAGGCAGGAAACCCC
FEL1m~R'	GGGGTTTCCTGCCTGTGATGCCCGTCTTCCAGCAAAGGGGTGC
FEL2m~F'	CCTGCAGGCCCTCTGCCTaGaTaGTCGGCCGAGACTGGAGGATC
FEL2m~R'	GATCCTCCAGTCTCGGCCGACtAtCtAGGCAGAGGGCCTGCAGG
FEL3m~F'	CCAACACGGGCTCAAACATGGGCAGCCCTGGACGGTGGGCATC
FEL3m~R'	GATGCCCACCGTCCAGGGCTGCCCATGTTTGAGCCCGTGTTGG
FEL4m~F'	GCTTCCTGCTGCCCTCTTACACTTGTAGCTCAGCTGCCTCTGGG
FEL4m~R'	CCCAGAGGCAGCTGAGCTACAAGTGTAAGAGGGCAGCAGGAAGC
FEL1Fdm	GACGGGCATCACAGGCAGCAAACCCCTGATTAAAGTACAAAG
FEL1Rdm	CTTTGTACTTTAATCAGGGGTTTGCTGCCTGTGATGCCCGTC
FEL3Fdm	CATGGGCAGCCCTGGACGGTGGGCATGCGGACTCCGGGTCCCATCG
FEL3Rdm	CGATGGGACCCGGAGTCCGCATGCCCACCGTCCAGGGCTGCCCATG
