# Supplementary material for: Identification of endothelial and mesenchymal FOXF1 enhancers involved in alveolar capillary dysplasia
Source: Nat Commun. 2024 Jun 19;15:5233. doi: 10.1038/s41467-024-49477-6 (PMC11187179; doi:10.1038/s41467-024-49477-6)
Supplement: Supplementary file 3 — Reporting Summary [file 41467_2024_49477_MOESM3_ESM.pdf]

nature portfolio

Corresponding author(s): Vladimir V. Kalinichenko
Guolun Wang

Last updated by author(s): Apr 29, 2024

Reporting Summary

Nature Portfolio wishes to improve the reproducibility of the work that we publish. This form provides structure for consistency and transparency in reporting. For further information on Nature Portfolio policies, see our Editorial Policies and the Editorial Policy Checklist.

Statistics

For all statistical analyses, confirm that the following items are present in the figure legend, table legend, main text, or Methods section.

n/a	Confirmed	
☐	☒	The exact sample size (*n*) for each experimental group/condition, given as a discrete number and unit of measurement
☐	☒	A statement on whether measurements were taken from distinct samples or whether the same sample was measured repeatedly
☐	☒	The statistical test(s) used AND whether they are one- or two-sided<br>*Only common tests should be described solely by name; describe more complex techniques in the Methods section.*
☒	☐	A description of all covariates tested
☒	☐	A description of any assumptions or corrections, such as tests of normality and adjustment for multiple comparisons
☐	☒	A full description of the statistical parameters including central tendency (e.g. means) or other basic estimates (e.g. regression coefficient) AND variation (e.g. standard deviation) or associated estimates of uncertainty (e.g. confidence intervals)
☐	☒	For null hypothesis testing, the test statistic (e.g. *F*, *t*, *r*) with confidence intervals, effect sizes, degrees of freedom and *P* value noted<br>*Give P values as exact values whenever suitable.*
☒	☐	For Bayesian analysis, information on the choice of priors and Markov chain Monte Carlo settings
☒	☐	For hierarchical and complex designs, identification of the appropriate level for tests and full reporting of outcomes
☒	☐	Estimates of effect sizes (e.g. Cohen's *d*, Pearson's *r*), indicating how they were calculated

Our web collection on statistics for biologists contains articles on many of the points above.

Software and code

Policy information about availability of computer code

Data collection	Cell Ranger ARC 1.0.0 – multiome Barcode Identification, Alignment, Filter, Demultiplex<br>Cell Ranger 7.0.0 – single cell RNA sequencing Barcode Identification, Alignment, Filter, Demultiplex<br>BD FACSdiva 9.0.1 was used to collect flow cytometry data.<br>NIS elements 5.25 was used to collect image data.
Data analysis	R version 4.1.2 – R environment for all R code<br>Seurat_4.0 – single-cell RNA data<br>Signac _1.7– single-cell chromatin data<br>other R packages:<br>ggpubr_0.3.0, ggplot2_3.3.0, uwot_0.1.8, reshape2_1.4.3, ggrepel_0.8.2,tsne_0.1-3,corrplot_0.84,magrittr_1.5,tidyr_1.0.2, limma_3.8.3, future_1.17.0, scales_1.1.0 , patchwork_1.0.0, dplyr_0.8.5, cowplot_1.0.0, corrplot_0.84, data.table_1.12.8, forcats_0.5.0, pheatmap_1.0.12,scrublet_2.01, scCustomize_2.12, igv genome browser_ 1.6<br>Flow cytometry data was analyzed by FlowJo V10.8.1.<br>Comparative genome analysis was completed by washU epigenomic browser<br>Genome analysis is performed using UCSC genome browser<br>Enhancer sequences were retrieved using UCSC genome browser.<br>Multiple alignments were performed with Ugene v42 was used to process genomic sequence.<br>Code used for the analysis of scRNA-seq data is available at the public Github repository at https://github.com/WGLUN/Foxf1multiome

For manuscripts utilizing custom algorithms or software that are central to the research but not yet described in published literature, software must be made available to editors and reviewers. We strongly encourage code deposition in a community repository (e.g. GitHub). See the Nature Portfolio guidelines for submitting code & software for further information.

Data

Policy information about availability of data

All manuscripts must include a data availability statement. This statement should provide the following information, where applicable:

- Accession codes, unique identifiers, or web links for publicly available datasets
- A description of any restrictions on data availability
- For clinical datasets or third party data, please ensure that the statement adheres to our policy

The single nuclei multiome data and single cell RNA seq data generated in this study have been deposited in the GEO database under accession code GSE217194. The single-cell RNA sequencing data for cells derived from WT and mutant mESCs via blastocyst complementation have been deposited in the GEO database under accession code GSE217194. https://www.ncbi.nlm.nih.gov/geo/query/acc.cgi?acc=GSE217194.
The single-cell ATACseq data of mouse E8.5 embryo are publicly available at GSE133244.
https://www.ncbi.nlm.nih.gov/geo/query/acc.cgi?acc=GSE133244. The bulk-ATAC data for mouse embryonic lung development were generated by ENCODE consortium, and the processed bigwig files are available at the GEO database with accession numbers GSE172744 (E14.5), GSE172933 (E15.5), GSE172813 (E16.5) and GSE172769 (E18.5).
https://www.ncbi.nlm.nih.gov/geo/query/acc.cgi?acc=GSE172744
https://www.ncbi.nlm.nih.gov/geo/query/acc.cgi?acc=GSE172933
https://www.ncbi.nlm.nih.gov/geo/query/acc.cgi?acc=GSE172813
https://www.ncbi.nlm.nih.gov/geo/query/acc.cgi?acc=GSE172769
The evolutionary conservation track data (phyloP and PhastCons) were downloaded from UCSC genome browser (https://genome.ucsc.edu). The human ACDMPV patient multiome datasets are publicly available at LungMAP data collection (https://data-browser.lungmap.net/projects). The FOXF1 Chipseq dataset was deposited to the public GEO database with accession number GSE77951, The EP300 ChIPseq dataset from mouse fetal lung was generated by ENCODE consortium and is publicly available at GSE91841. The EP300 ChIPseq data from mouse adult lung is available to GEO database (GSE88789). https://www.ncbi.nlm.nih.gov/geo/query/acc.cgi?acc=GSE77951
https://www.ncbi.nlm.nih.gov/geo/query/acc.cgi?acc=GSE91841
https://www.ncbi.nlm.nih.gov/geo/query/acc.cgi?acc=GSE88789

Research involving human participants, their data, or biological material

Policy information about studies with human participants or human data. See also policy information about sex, gender (identity/presentation), and sexual orientation and race, ethnicity and racism.

Reporting on sex and gender	The human data used in this study were published multiome sequencing in ACDMPV patient lungs (PMID:37463497). The sex and gender reported in that publication.
Reporting on race, ethnicity, or other socially relevant groupings	The human data used in this study were published multiome sequencing in ACDMPV patient lungs (PMID:37463497). The race and ethnicity were reported in that publication.
Population characteristics	*Describe the covariate-relevant population characteristics of the human research participants (e.g. age, genotypic information, past and current diagnosis and treatment categories). If you filled out the behavioural & social sciences study design questions and have nothing to add here, write "See above."*
Recruitment	*Describe how participants were recruited. Outline any potential self-selection bias or other biases that may be present and how these are likely to impact results.*
Ethics oversight	*Identify the organization(s) that approved the study protocol.*

Note that full information on the approval of the study protocol must also be provided in the manuscript.

Field-specific reporting

Please select the one below that is the best fit for your research. If you are not sure, read the appropriate sections before making your selection.

☒ Life sciences ☐ Behavioural & social sciences ☐ Ecological, evolutionary & environmental sciences

For a reference copy of the document with all sections, see nature.com/documents/nr-reporting-summary-flat.pdf

Life sciences study design

All studies must disclose on these points even when the disclosure is negative.

Sample size	No statistical method to predetermine sample size. For flow cytometry experiments, we chose to analyze 6 animals /group in order to improve the reproducibility which meet the current standard in the field.
Data exclusions	In single cell experiments, the cells which didn't pass the quality check were excluded from the analysis in an unbiased way. No other methods were used to exclude cell data.

Replication

Lung from 6 different mice in the same litter were pooled to make single cell multimode sequencing library. The RNA library (GEX) in the library was validated with previously published single cell RNA seq data generated using the same FACS protocol.
For luciferase assay, the transfections in each condition were carried out in 4 times. The results were reproducible.
For blastocyst complementation, experiments, each reported results were achieved 3 times.

Randomization

All mice used in the same experiment were randomized to avoid bias.
For flow cytometry experiments, lung samples were randomly grouped and cells were randomly grouped and stained for analysis.
For blastocyst complementation, all blastocyst collected from different mouse were pooled and then randomly grouped for microinjection. After incubation, the survived blastocysts were randomly selected based on grouping of injection of different cell types for implanting.

Blinding

Lung cells consisted of 4 main groups, epithelial, stromal, endothelial and hematopoietic cells. We depleted the hematopoietic cells, epithelial cells and stromal cells no Foxf1 expression using flow cytometry before preparing library for single nuclei multiome seqeuncing. When preparing the library for sequencing, 12000 cells were loaded to flowcell, and ~6000 cells of good quality were recovered. Sample size was selected to make sure that the total number of sequenced cell was sufficient to examine both major and rare cell populations.
The single cell multiome sequencing, RNA sequencing were carried out following the standard protocol. The following bioinformatic analysis were performed in fully unbiased manner.For flow cytometry analysis and blastocyst complementation, researchers were blinded to group determination, data collection and data analysis.
For in vitro (non-sequencing) experiments, no blinding was required because experiments were designed with biological replicates and technical replicates to avoid bias. For microscopy experiments in lung tissues, the researchers performing the staining and analysis were blinded.

Reporting for specific materials, systems and methods

We require information from authors about some types of materials, experimental systems and methods used in many studies. Here, indicate whether each material, system or method listed is relevant to your study. If you are not sure if a list item applies to your research, read the appropriate section before selecting a response.

Materials & experimental systems

n/a	Involved in the study
☐	☒ Antibodies
☐	☒ Eukaryotic cell lines
☒	☐ Palaeontology and archaeology
☐	☒ Animals and other organisms
☒	☐ Clinical data
☒	☐ Dual use research of concern
☒	☐ Plants

Methods

n/a	Involved in the study
☒	☐ ChIP-seq
☐	☒ Flow cytometry
☒	☐ MRI-based neuroimaging

Antibodies

Antibodies used

(CD31,1:100,eFluor450,FC,Thermofisher,48-0311-82);
(CD45,1:100,APC-eFluor780,FC,Thermofisher,47-0451-82);
(CD140a,1:100,PE-Cy7,FC,Thermofisher,25-1401-82);
(CD146),1:100,PerCP,FC,Biolegend,134710);
(CD45,1:100,AF700,FC,Thermofisher,56-0451-82);
(CD31,1:100,BV605,FC,Biolegend,102427);
(CD326,1:100,APC,FC,Biolegend,118212);
(TruStain FcX, 1:100, NA, Biolegend, 101320);

Validation

*CD31-Thermofisher-48-0311-82 Supplier's webpage This antibody proved to detect endogenouse Pecam1 in flow cytometry https://www.thermofisher.com/antibody/product/CD31-PECAM-1-Antibody-clone-390-Monoclonal/48-0311-82
*CD45-Thermofisher-47-0451-82 Supplier's webpage This antibody proved to detect endogenouse Ptprc in flow cytometry https://www.thermofisher.com/antibody/product/CD45-Antibody-clone-30-F11-Monoclonal/47-0451-82
*CD140a-Thermofisher-25-1401-82 Supplier's webpage This antibody proved to detect endogenouse Pdgfra in flow cytometry https://www.thermofisher.com/antibody/product/CD140a-PDGFRA-Antibody-clone-APA5-Monoclonal/25-1401-82
*CD146-Biolegend-120413 Supplier's webpage This antibody proved to detect endogenouse Mcam in flow cytometry https://www.biolegend.com/en-us/products/percp-cyanine5-5-anti-mouse-cd146-antibody-7872
*CD45-Thermofisher-56-0451-82 Supplier's webpage This antibody proved to detect endogenouse Ptprc in flow cytometry https://www.thermofisher.com/antibody/product/CD45-Antibody-clone-30-F11-Monoclonal/56-0451-82
*CD31-Biolegend-102427 Supplier's webpage This antibody proved to detect endogenouse Pecam1 in flow cytometry https://www.biolegend.com/en-us/products/brilliant-violet-605-anti-mouse-cd31-antibody-9963
*CD326-Biolegend-118212 Supplier's webpage This antibody proved to detect endogenouse Epcam in flow cytometry https://www.biolegend.com/en-us/products/alexa-fluor-647-anti-mouse-cd326-ep-cam-antibody-4973
TruStain FcX-101320, supplier's webpage This antibody proved to useful for blocking non-specific binding of immunoglobulin to the Fc receptors in flow cytometry https://www.biolegend.com/en-us/products/trustain-fcx-anti-mouse-cd16-32-antibody-5683

Eukaryotic cell lines

Policy information about cell lines and Sex and Gender in Research

Cell line source(s)	The MFLM-91U (AMFLM-91U) cell line was derived from mouse fetal lung and maintained by Sevenhills bioreagents. https://www.sevenhillsbioreagents.com/products/mflm-91u-cell-line
Authentication	The MFLM-91U cell line were obtained by Sevenhills bioreagets and were not further authenticated. Cells express proteins associated with endothelial cell phenotype including CD34, PECAM-1, vonWillebrand factor, VEGFR1 (flt1), VEGFR2 (flk-1) and cell-surface recognition site for lectin GSL B4. By PCR analysis, MFLM-91U have mRNA transcripts for Tie-1, Tie-2, Ang-1 and Ang-2. The cells also express vimentin and low levels of smooth muscle alpha actin. MFLM-91U take up acetylated LDL and on Matrigel form extensive multi-cell, tubular networks.
Mycoplasma contamination	The MFLM-91U cell line is tested negative of mycoplasma contamination.
Commonly misidentified lines (See ICLAC register)	The MFLM-91U cell line used in the study is not listed in the register.

Animals and other research organisms

Policy information about studies involving animals; ARRIVE guidelines recommended for reporting animal research, and Sex and Gender in Research

Laboratory animals	The mice used in multiome sequencing study were Foxf1-GFP reporter, which was generated in the genetic background of C57BL/6. We harvest the mice to collect embryos of E18.5. All embryos in a litter was collected, and we didn't determine the sex of the embryos.<br>The mice used in blastocyst complementation were CD1, adult mice.<br>All animal experiments were approved by the Institutional Animal Care and Use Committee of Cincinnati Children's Research Foundation. All mice were kept under SPF (specific-pathogen-free) conditions in 12/12 light/dark cycle, 18-24°C and 40-60% humidity.
Wild animals	No wild animals were used.
Reporting on sex	The sex of mouse embryos were not determined prior to harvesting the samples.
Field-collected samples	No field collected samples
Ethics oversight	All animal procedures were reviewed and approved by the Institutional Animal Care and Use Committee of Cincinnati Children's Research Foundation.

Note that full information on the approval of the study protocol must also be provided in the manuscript.

Plants

Seed stocks	*Report on the source of all seed stocks or other plant material used. If applicable, state the seed stock centre and catalogue number. If plant specimens were collected from the field, describe the collection location, date and sampling procedures.*
Novel plant genotypes	*Describe the methods by which all novel plant genotypes were produced. This includes those generated by transgenic approaches, gene editing, chemical/radiation-based mutagenesis and hybridization. For transgenic lines, describe the transformation method, the number of independent lines analyzed and the generation upon which experiments were performed. For gene-edited lines, describe the editor used, the endogenous sequence targeted for editing, the targeting guide RNA sequence (if applicable) and how the editor was applied.*
Authentication	*Describe any authentication procedures for each seed stock used or novel genotype generated. Describe any experiments used to assess the effect of a mutation and, where applicable, how potential secondary effects (e.g. second site T-DNA insertions, mosiacism, off-target gene editing) were examined.*

Flow Cytometry

Plots

Confirm that:

☒ The axis labels state the marker and fluorochrome used (e.g. CD4-FITC).

☒ The axis scales are clearly visible. Include numbers along axes only for bottom left plot of group (a 'group' is an analysis of identical markers).

☒ All plots are contour plots with outliers or pseudocolor plots.

☒ A numerical value for number of cells or percentage (with statistics) is provided.

Methodology

Sample preparation	The mouse lungs were perfused through the right ventricle with 5 ml of PBS to remove blood. The lung tissue was enzymatically digested using 0.2 mg/ml Liberase TM (Roche) and 100U/ml Deoxyribonuclease I (Sigma, DN25) for 30 mins, and then passed through cell strainer (70 µm pores) to obtain a single cell suspension. Red blood cells were lysed in ACK lysis buffer. To remove cell debris, single cell suspensions from the lung tissue were passed through cell strainer snap cap (Corning life sciences). Single cell suspensions were stained with fixable viability dye (Biolegend) followed by the incubation with TruStain FcX (Biolegend). To identify cell surface antigens, cells were stained with a mixture of fluorochrome-conjugated antibodies.
Instrument	BD Fusion and Fortessa
Software	BD FACSdiva 9.0.1
Cell population abundance	Cell population is easily identified with regular gating strategy.
Gating strategy	The gating strategy:<br>1) FSC-A/SSC-A for all cells.<br>2) SSC-W/SSC-H for singlets<br>3) FSC-H/FSC-W for singlets<br>4) Viability / FSC-A for live cells<br>5) CD31/CD45 for endothelial cells<br>6) Foxf1-GFP for FOXF1 expression

☒ Tick this box to confirm that a figure exemplifying the gating strategy is provided in the Supplementary Information.
